# Determination of oligosaccharide product distributions of PL7 alginate lyases by their structural elements

**DOI:** 10.1038/s42003-022-03721-1

**Published:** 2022-08-02

**Authors:** Keke Zhang, Zhijian Li, Qiaoyun Zhu, Huansheng Cao, Xinxin He, Xiao-Hua Zhang, Weizhi Liu, Qianqian Lyu

**Affiliations:** 1 MOE Key Laboratory of Marine Genetics and Breeding, College of Marine Life Sciences, Ocean University of China, Qingdao, China; 2 Laboratory for Marine Biology and Biotechnology, Pilot National Laboratory for Marine Science and Technology, Qingdao, China; 3 Division of Natural and Applied Sciences, Duke Kunshan University, Kunshan, China

**Keywords:** Biocatalysis, X-ray crystallography

## Abstract

Alginate lyases can be used to produce well-defined alginate oligosaccharides (AOSs) because of their specificities for AOS products. A large number of alginate lyases have been recorded in the CAZy database; however, the majority are annotated-only alginate lyases that include little information on their products, thus limiting their applications. Here, we establish a simple and experiment-saving approach to predict product distributions for PL7 alginate lyases through extensive structural biology, bioinformatics and biochemical studies. Structural study on several PL7 alginate lyases reveals that two loops around the substrate binding cleft determine product distribution. Furthermore, a database containing the loop information of all annotated-only single-domain PL7 alginate lyases is constructed, enabling systematic exploration of the association between loop and product distribution. Based on these results, a simplified loop/product distribution relationship is proposed, giving us information on product distribution directly from the amino acid sequence.

## Abstract (web summary)

Characterization of PL7 alginate lyase structure and products enables a bioinformatics approach to predict product distribution from the amino acid sequence.

## Introduction

Alginates are the most abundant polysaccharides in brown seaweeds, accounting for one-third of the polysaccharides in the ocean. These linear alginates consist of β-D-mannuronic acid (M) and its C-5 epimer α-L-guluronic acid (G), and the two uronates are linked by 1,4-glycosidic bonds arranging into homopolyuronic blocks of M residues (polyM) and G residues (polyG) or heteropolyuronic blocks (polyMG or polyGM)^1^. In heterotrophic bacteria that use alginates as crucial nutrients, alginate lyases depolymerize alginate into alginate oligosaccharides (AOSs) and then into monomers for assimilation into the central metabolism^2,3^. Accordingly, the degree of polymerization (Dp) of the generated AOSs can be used to analyze the bacterial utilization of alginate. More importantly, AOSs have demonstrated attractive biological activities, including antioxidant^4^, antibacterial^5^, antitumor^6^, and immune regulation activities^7^, which have been proven to be influenced by the Dp of AOSs^8,9^. For example, the antitumor activity of AOSs in osteosarcoma cells has been examined using four AOS types (Dp = 2, Dp = 3, Dp = 4 and Dp = 5), but only the AOS with Dp5 displays efficacy^10^. Obviously, the Dp of AOSs is determined by alginate lyase specificity (here, we name this specificity Dp specificity to distinguish it from substrate recognition specificity), and knowledge of alginate lyase Dp specificity facilitates not only an understanding of the metabolic processes of alginates but also, and more importantly, the artificial preparation of well-defined AOSs^11,12^.

The 14 polysaccharide lyase (PL) families are classified based on the amino acid sequences of the alginate lyases (PLs 5, 6, 7, 8, 14, 15, 17, 18, 31, 32, 34, 36, 39, and 41, CAZy database, http://www.cazy.org/); among these, PL7 is the largest and most extensively studied family^12–14^. The PL7 family contains a total of 2187 alginate lyases, of which only 44 have been experimentally characterized as of May 2021. In particular, with the increasing number of genomic studies on marine organisms, an increasing number of alginate lyase genes will inevitably be discovered, and hence, the gap between annotated-only alginate lyases (i.e., those with only amino acid sequence information available) and the characterized alginate lyases will increase. Obviously, these annotated-only alginate lyases are potential candidates for AOS preparation. However, the question of how to understand/use annotated-only alginate lyases efficiently arises, which requires us to explore an approach to quickly understand annotated-only alginate lyases, especially their Dp specificities.

Based on the action mode, alginate lyases are generally grouped into exolytic alginate lyases and endolytic alginate lyases. Cleavage by exolytic alginate lyases occurs at the end of the substrate, resulting in monomer release after every reaction and clear Dp specificity (i.e., monomer specificity)^15^. In contrast, endolytic alginate lyases perform cleavage reactions randomly within the substrate, resulting in a mixture of oligomeric products with different Dps^16^. It seems that the Dp specificities of endolytic alginate lyases are difficult to define and compare because all of these lyases generate oligomer mixtures. However, we found that endolytic alginate lyases show different Dp specificities if the quantity of each oligosaccharide in the mixture of products is taken into account. For example, the products of alginate lyases AlyH1 and AlgSH7 both ranged from Dp2 to Dp4, of which the most abundant products were disaccharides and trisaccharides, respectively, showing Dp2 specificity for AlyH1 but Dp3 specificity for AlgSH7^17,18^. Thus, endolytic alginate lyases can be further classified based on their Dp specificities if the relationship between Dp specificity (or product distribution) and amino acid sequence is determined. Thus, such a classification will provide a quick guide for understanding the Dp specificities of AOS products without carrying out experiments, which is of great significance to understand the roles of annotated-only alginate lyases in the marine carbon cycle and in the preparation of well-defined AOSs^19^. To further divide endolytic alginate lyases, the amino acid sequence/Dp specificity relationship of alginate lyase should be elucidated, and to this end, the three-dimensional structure/Dp specificity relationship (i.e., structure/Dp specificity relationship) of alginate lyase should first be determined. Considering that PL7 is the largest and most extensively studied family of alginate lyases, it is the most suitable candidate for exploring the structure/Dp specificity relationship. Thus, we focused on PL7 alginate lyases in this study.

As of May 2021, 12 alginate lyase structures have been solved (PDB: 1VAV^20^, 1UAI^21^, 5XNR^22^, 5ZQI, 3ZPY^23^, 4BE3^23^, 4OZX, 5ZU5^24^, 5Y33^25^, 7C8G/7C8F^26^, 2CWS/2ZAA^27,28^ and 6YWF), including two structures of alginate lyase-oligosaccharide complex. The overall structures of these PL7 alginate lyases are similar, showing a β-jelly roll fold composed of two antiparallel β-sheets with a deep cleft. The noteworthy structural differences among the solved PL7 structures are the loops around the deep cleft, which are variable in length and sequence and involved in alginate substrate binding with the deep cleft. To explore the structure/Dp specificity relationship of PL7 alginate lyases, the corresponding substrate binding mechanism should first be clarified. Determining the structure of alginate lyase A1-II’-GGMG complex elucidated the substrate binding profile at subsites −1, +1, +2, and +3, revealing some important interactions formed between the alginate lyase and the substrate^27^. However, substrate binding information is still lacking because of the possible existence of subsites −2 and −3. Therefore, determination of more structures of alginate lyase-oligosaccharide complex is required to reveal the key structural elements that influence the Dp of AOS products.

Here, multidisciplinary techniques were applied to establish the relationship between product distribution and amino acid sequence, including structural biology, bioinformatics and biochemical methods. First, we demonstrated that two loops (loop1 and loop2) at the “-” region of the substrate binding cleft of single-domain PL7 alginate lyases are the determinant for their product distributions. Furthermore, the length of loop1 could be used to evaluate the product distribution. Next, the detailed correlation between the length of loop1 and product distribution was explored at the level of the whole PL7 family in combination with the loop1 sequence determination approach. Therefore, the loop/product distribution relationship for annotated-only single-domain PL7 alginate lyases was summarized as follows: an alginate lyase with a long loop1 (loop1 length>11) tends to generate trisaccharides and disaccharides, whereas an alginate lyase with a short loop1 (loop1 length ≤11) tends to generate larger oligosaccharides (Dp of the product >3). These findings provide an experiment-saving strategy to quickly understand annotated-only PL7 alginate lyases, which might promote the usage of these alginate lyases in the preparation of well-defined AOSs.

## Results and discussion

### Two PL7 alginate lyases with different product distributions, AlyV and PyAly, were selected as reference enzymes

**Fig. 1:**
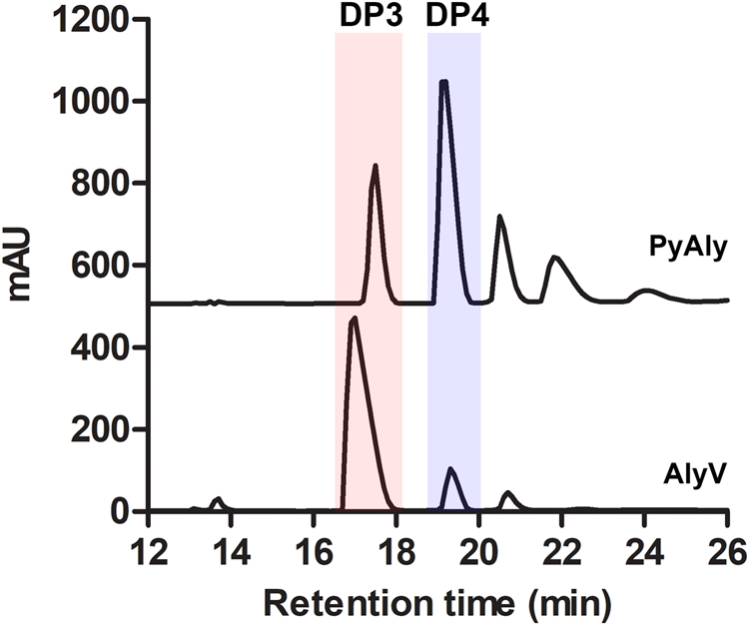
Product distributions of PyAly and AlyV. The products generated by PyAly and AlyV were analyzed by high-performance liquid chromatography (HPLC). The main products of AlyV and PyAly were trisaccharides and tetrasaccharides, respectively, showing different Dp specificities.

To explore the structural elements that affect product distribution and hence Dp specificity, alginate lyases that would be suitable for comparison analysis were first screened. AlyV is a PL7 alginate lyase identified in this study that preferentially degrades polyM when presented with alginate, polyG and polyM as substrates (the detailed enzymatic properties of AlyV are presented in Supplementary Fig. 1a, Supplementary Fig. 2, Supplementary Table 1). In addition, it was reported that the PL7 alginate lyase PyAly also shows substrate specificity for polyM (Supplementary Fig. 1b)^29^. However, the Dp specificities of AlyV and PyAly are different. As shown in Fig. 1 and Supplementary Table 2, AlyV mainly degrades polyM into trisaccharides, with a trisaccharide content of approximately 82.9%. For the product generated by PyAly, the trisaccharide content was approximately 21.9% whereas the tetrasaccharide content was 44.8%, making tetrasaccharides the main product. The difference in product distributions can be attributed to their different substrate binding profiles. Accordingly, elucidation of the substrate binding profile differences between them would contribute to exploring the structural element functions in product distribution. Therefore, the structures of AlyV/PyAly-oligosaccharide complex were solved and compared (see below).

**Fig. 2:**
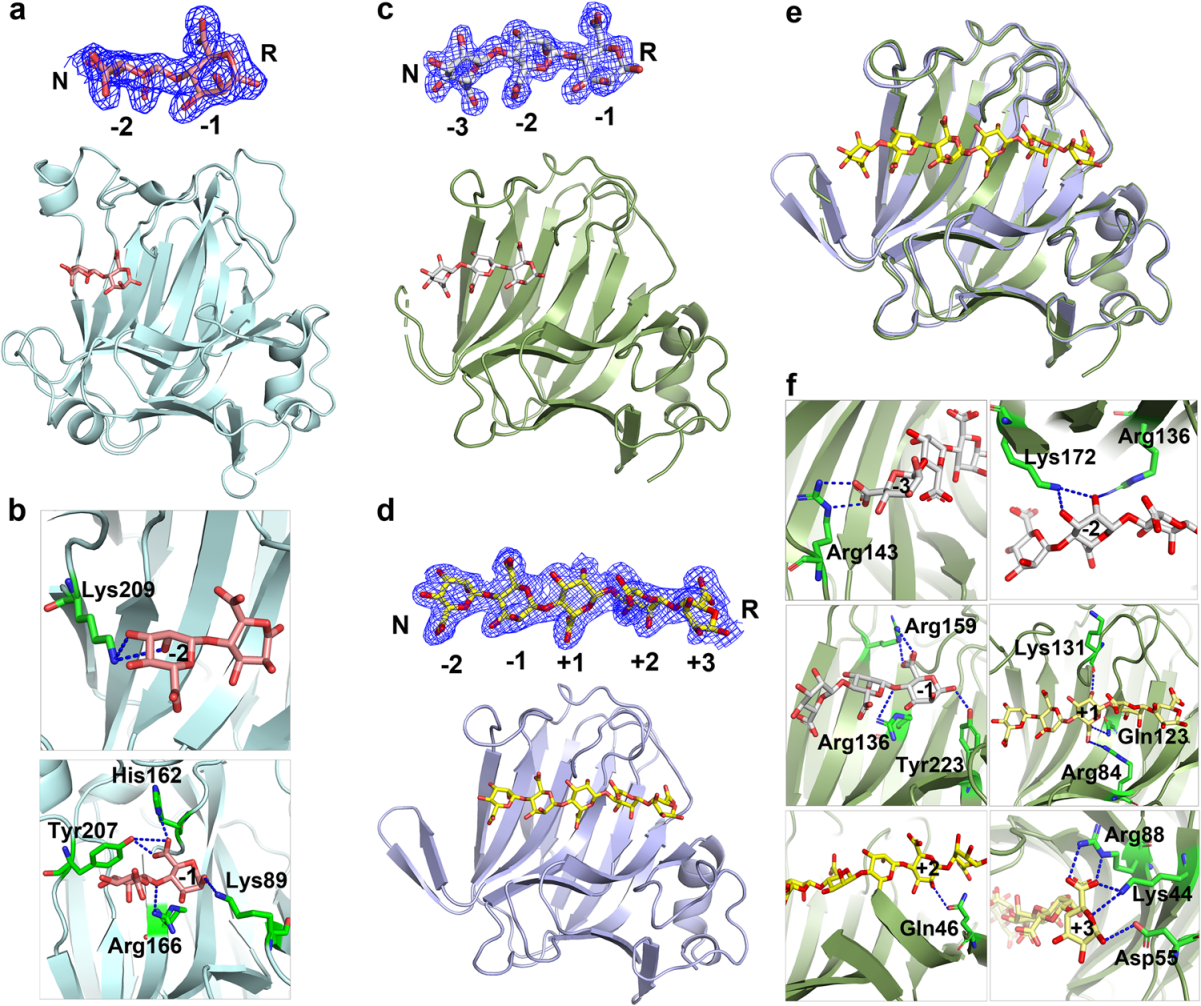
Structural analysis of the AlyV^R91A^-M8, PyAly^H125A^-M8 and PyAly^H125A_Y223A^-M5 complexes. Overall structures of the **a** AlyV^R91A^-M8 complex, **c** PyAly^H125A^-M8 complex and **d** PyAly^H125A_Y223A^-M5 complex. AlyV^R91A^, PyAly^H125A^ and PyAly^H125A_Y223A^ are shown as cartoons in pale cyan, dark green and light blue, respectively. M8 and M5 are shown as sticks in salmon **a**, gray **c**, and yellow **d**, respectively. The electron density maps for M8 and M5 (contour level 1σ) are shown above the overall structures of the complexes. **b** Hydrogen bonding interactions between AlyV^R91A^ and the sugar units at subsites -1 and -2. The residues involved in substrate binding are shown in sticks, and hydrogen bonds are indicated with blue dashed lines. **e** Structural comparison of the PyAly^H125A^-M8 complex with the PyAly^H125A_Y223A^-M5 complex. **f** Hydrogen bonding interactions between PyAly and the sugar units at subsites -3 to +3. The residues involved in substrate binding are shown in stick, and hydrogen bonds are indicated with blue dashed lines.

To obtain information about the substrate binding cleft of AlyV, the oligomeric substrate M8 (MMMMMMMM) was cocrystallized with inactive AlyV mutants. Three AlyV mutants (AlyV^Q139A^, AlyV^H141N^, and AlyV^Y261F^) were designed and prepared based on the conserved His (Tyr’)/Tyr β-elimination catalytic mechanism in PL7 alginate lyases; that is, His141 and Tyr261 act as the general base and acid, respectively, and Gln139 acts to neutralize the C-5 carboxyl group^30^. Additionally, the AlyV^R91A^ mutant was prepared because of the conserved Arg91 residue in PL7 alginate lyases and the substate binding function of Arg146 (corresponding to Arg91 in AlyV) as determined in the structure of A1-II’-GGMG complex (PDB: 2ZAA)^27,30^. Then, substrate M8 was cocrystallized with the four AlyV mutants, resulting in the corresponding structures. Unfortunately, no sugar unit was observed in the structures of the AlyV^Q139A^-, AlyV^H141N^- and AlyV^Y261F^-M8 complexes, and only two sugar units were observed in the structure of the AlyV^R91A^-M8 complex, which was attributed to the minor residual catalytic activity of the AlyV mutants. The crystal structure of the AlyV^R91A^-M8 complex was solved at 1.90 Å, showing the typical β-jelly roll fold with two sugar units bound at subsites -1 and -2 (Fig. 2a, Table 1). The interactions between AlyV^R91A^ and the two sugar units are presented in Supplementary Fig. 3, and the key hydrogen bonds are described below (Supplementary Table 3).

**Table 1:** Summary of data processing and refinement statistics.

Data collection	PyAly^H125A^-M8	PyAly^H125A_Y223A^-M5	AlyV^R91A^-M8	AlyB^H360A_Y466A^-G9
PDB ID Space group	7W13 *P* 2_1_2_1_2_1_	7W18 *P3*_*1*_*2*_*1*_	7W16 *P3*_*1*_*2*_*1*_	7W12 *P 2*_*1*_*2*_*1*_*2*_*1*_
Cell dimensions, *a*,*b*,*c* (Å) Cell dimensions, *α,β,γ* (°)	47.3, 64.5, 73.8 90.0, 90.0, 90.0	63.1, 63.1, 114.5 90.0, 90.0, 120.0	80.4, 80.4, 113.9 90.0, 90.0, 120.0	48.8, 67.3, 78.2 90.0,90.0, 90.0
X-ray source	SSRF BL 18U	SSRF BL 19U	SSRF BL 18U	SSRF BL 18U
Wavelength (Å)	0.9785	0.9785	0.9785	0.9785
Resolution (Å)^a^	50–1.62 (1.65–1.62)	50–1.7 (1.73–1.70)	50–1.9 (1.97–1.9)	50–2.25 (2.29–2.25)
Unique reflections^a^	29611	28715	34336	40570
Completeness (%)	99.9 (100)	100 (99.1)	100 (100)	100 (100)
Mean(I/σ)^a^	30.4 (4.6)	21.1 (2.3)	20.7 (3.7)	45 (4.3)
CC1/2	0.993 (0.940)	0.998 (0.893)	1.002 (0.896)	0.998 (0.917)
Redundancy^a^	12.8 (7.8)	18.3 (15.8)	17.8 (15.6)	14.6 (13.8)
*Refinement statistics*				
Resolution (Å)^a^	40–1.65 (1.69–1.65)	50–1.70 (1.74–1.70)	50–1.90 (1.95–1.90)	50–2.25(2.31–2.25)
*R*_factor_ (%)	20.9 (12.4)	24.8 (29.7.)	14.5 (18.3)	19.6 (23.8)
Free *R*_factor_ (%)	24.1 (27.4)	28.5 (31.8)	19.3 (27.7)	23.4 (31.6)
Residues built (range) total	Chain A:27-241	Chain A:27-241	Chain A:25-287	Chain A:4-493
Free *R* reflections (%)	5.0	5.1	5.1	5.0
Free *R* reflections no.	1398	1327	1706	2017
No. non-hydrogen atoms	1796	1864	2229	4011
*Model quality*				
RMSD bond length (Å)	0.023	0.030	0.022	0.017
RMSD bond angles (°)	2.11	2.80	2.31	1.84
Mean B-factors				
Overall (Å^2^)	14.2	13.8	19.3	31.8
Protein atoms (Å^2^)	14.2	14.9	21.8	32.6
Water (Å^2^)	18.5	23.8	30.3	28.4
ligand (Å^2^)	20.7	17.3	27.7/33.1	37.3/52.1
Ramachandran plot (%), favored/allowed/disallowed	97.3/2.7/0	94.2/5.8/0	98.0/2.0/0	97.0/3.0/0

^a^Parentheses indicate the highest resolution.

*-2 subsite*: The side chain of Lys209 formed two hydrogen bonds with the hydroxyl groups at the C-2 and C-3 positions (Fig. 2b).

*-1 subsite*: The side chain of Tyr207 formed two hydrogen bonds with the C-5 carboxyl group, and the side chain of His162 formed one hydrogen bond with the C-5 carboxyl group. The side chain of Lys89 formed one hydrogen bond with the hydroxyl group at the C-2 position. Additionally, the side chain of Arg166 interacted with the glycosidic oxygen from C-4 (Fig. 2b).

Similar to AlyV, several PyAly mutants and two oligomeric substrates (M8 and M5) were used to determine the structures of PyAly-oligosaccharide complexes. The structures of the PyAly^H125A^-M8 complex and the PyAly^H125A_Y223A^-M5 complex were solved at 1.62 Å and 1.70 Å, respectively (Fig. 2c, d, Table 1). Except for the sugar units bound at subsites −3, −2, and −1, the other sugar units of M8 were not observed in the structure of the PyAly^H125A^-M8 complex, which was similar to the observation in the structure of the AlyV^R91A^-M8 complex. In contrast, the structure of the PyAly^H125A_Y223A^-M5 complex presented the complete M5 substrate at subsites −2, −1, +1, +2, and +3. Through structure alignment between the PyAly^H125A^-M8 complex and the PyAly^H125A_Y223A^-M5 complex, the overall sugar binding profile from subsites -3 to +3 is presented in Fig. 2e.

According to the abovementioned two structures, the interactions between PyAly and the six sugar units are presented in Supplementary Fig. 4, and the key hydrogen bonds are described below (Supplementary Table 4).

*-3 subsite*: The side chain of Arg143 formed two hydrogen bonds with the C-5 carboxyl group (Fig. 2f).

*-2 subsite*: The side chain of Lys172 formed hydrogen bonds with the hydroxyl groups at the C-2 and C-3 positions. The side chain of Arg136 formed one hydrogen bond with the hydroxyl group at the C-2 position (Fig. 2f).

*-1 subsite*: The side chain of Arg159 formed two hydrogen bonds with the C-5 carboxyl group. The side chain of Tyr223 formed one hydrogen bond with the hydroxyl group at the C-1 position. The side chain of Arg136 interacted with the glycosidic oxygen at C-4 (Fig. 2f).

*+1 subsite*: The side chain of Arg84 formed two hydrogen bonds with the C-5 carboxyl group, and the side chain of Gln123 formed one hydrogen bond with the C-5 carboxyl group. The side chain of Lys131 formed one hydrogen bond with the hydroxyl group at the C-2 position (Fig. 2f).

*+2 subsite*: The side chain of Gln46 formed one hydrogen bond with the hydroxyl group at the C-2 position (Fig. 2f).

*+3 subsite*: The side chain of Arg88 formed two hydrogen bonds with the C-5 carboxyl group, and the side chain of Lys44 formed one hydrogen bond with the C-5 carboxyl group. Additionally, the side chain of Lys44 interacted with the O atom of the sugar ring. The side chain of Asp55 formed one hydrogen bond with the hydroxyl group at the C-1 position (Fig. 2f).

**Fig. 3:**
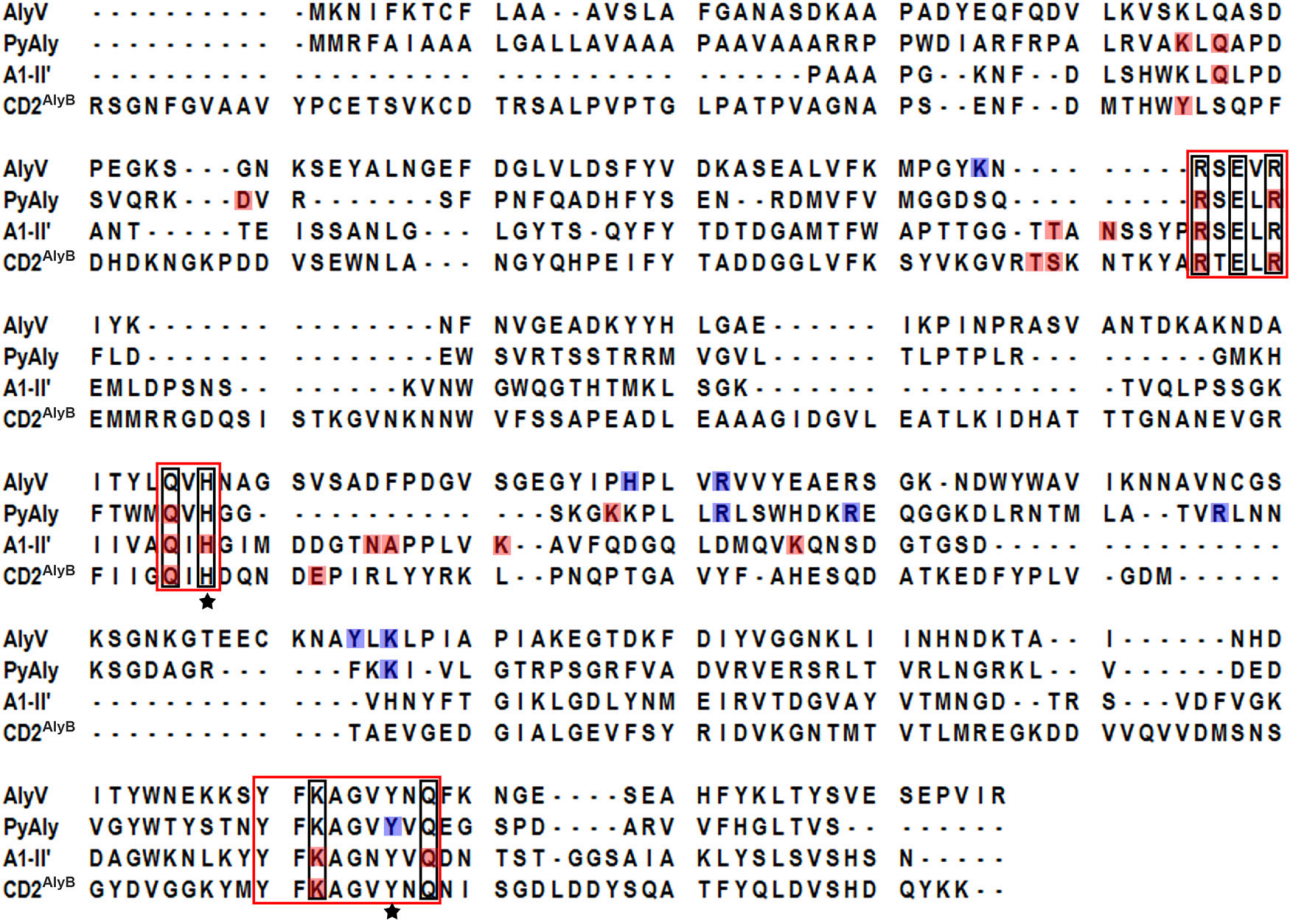
Sequence alignment of AlyV, PyAly, A1-II’ and CD2^AlyB^. The three conserved regions in the PL7 family are boxed in red, and two conserved catalytic residues are marked with stars. For PyAly, A1-II’ and CD2^AlyB^, the residues involved in substrate binding at subsites +1 to +3 are shaded in red, and the conserved residues in the PL7 family are boxed in black. For PyAly and AlyV, the residues involved in substrate binding at subsites -1 to -3 are shaded in blue.

Interestingly, comparison of the PyAly, AlyV, and A1-II’ residues involved in substrate binding showed that most of the PyAly residues functioning in subsites +1 to +3 were conserved in AlyV and A1-II’, whereas the PyAly residues functioning in subsites −1 to −3 shared low sequence identity with AlyV and A1-II’ (Fig. 3). Notably, the conserved residues acting in subsites +1 to +3 were mainly located in three regions: QI(V)H, RXEL(V)R, and YFKXGXYXQ, which are also conserved throughout the PL7 family, suggesting possible similar substrate binding at subsites +1 to +3 for all PL7 alginate lyases^31^.

### Extensive substrate binding cleft analysis revealed that substrate binding at the “-” section of single-domain PL7 alginate lyases might determine product distribution

**Fig. 4:**
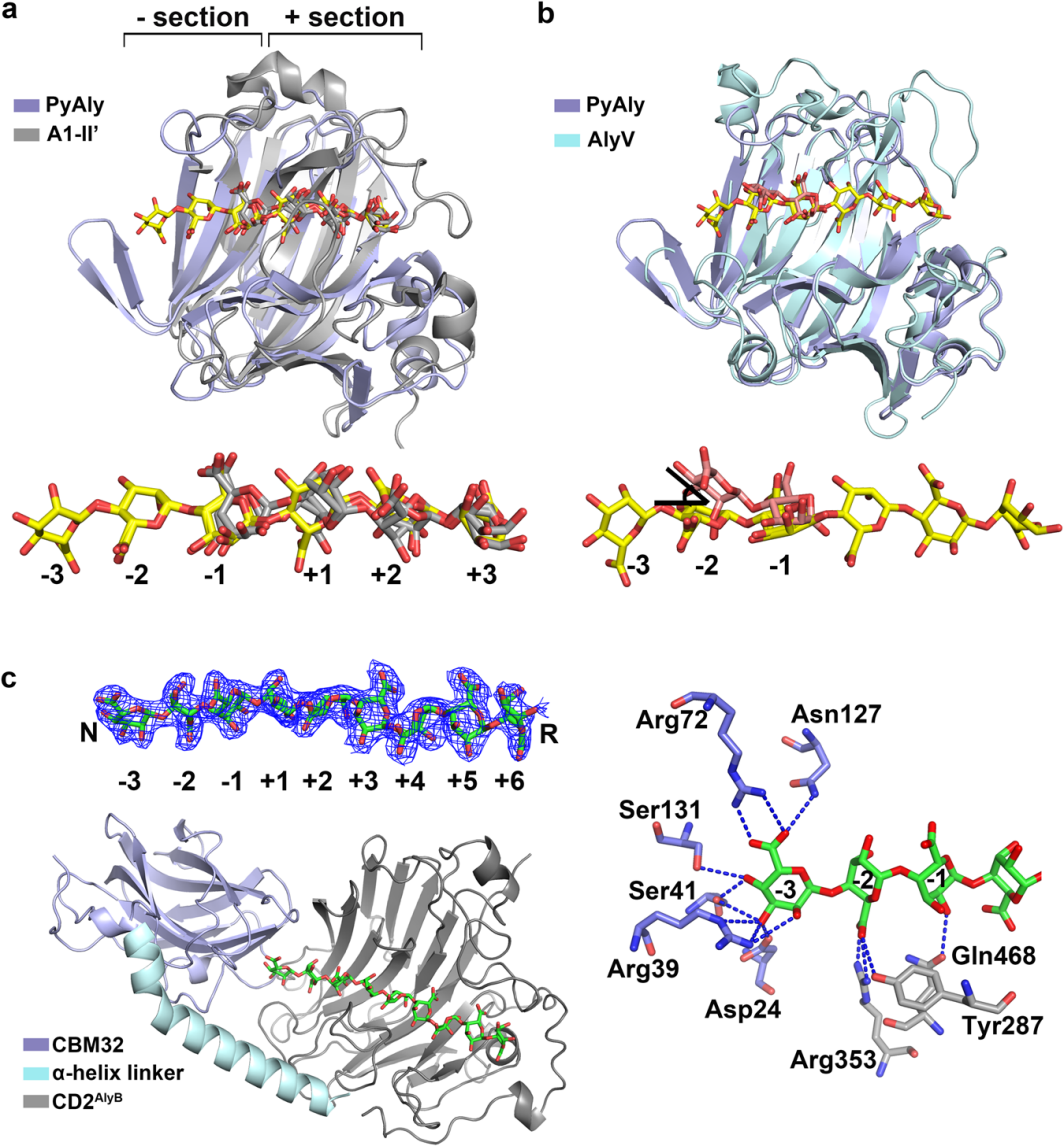
Comparative analysis of the substrate binding profiles of PyAly, A1-II’, AlyV and AlyB revealed the relationship between the “-” section and product distribution. **a** Structural comparison of the PyAly^H125A^-M8 + M5 complex and the A1-II’-GGMG complex. The sugars bound in PyAly and A1-II’ are shown as sticks in yellow and gray, respectively. The substrate binding clefts of PL7 alginate lyases were divided into “+” sections and “-” sections using the cleavage site as the boundary. Similar substrate binding profiles at the “+” sections were observed. **b** Structural comparison of the PyAly^H125A^-M8 + M5 and AlyV^R91A^-M8 complexes. The sugar bound in AlyV is shown in salmon. A clear angle was observed between the two sugar units at subsites -2, indicating different substrate binding profiles in the “-” sections. **c** Structural analysis of the AlyB^H360A_Y466A^-G9 complex. AlyB is shown in cartoon, and G9 is shown in green. The electron density map for G9 (contour level 1σ) is presented. The residues that formed hydrogen bonding interactions with subsites -3 to -1 are shown as sticks: the residues from CBM32 are in slate, and the residues from CD2^AlyB^ are in gray. Hydrogen bonds are indicated with blue dashed lines.

The determination of structures for PyAly/AlyV-oligosaccharide complex allowed us to gain detailed structural insights into the substrate binding cleft. First, a structural comparison of the PyAly^H125A^-M8 + M5 complex and the A1-II’-GGMG complex was carried out. As expected, the substrate binding clefts of PyAly and A1-II’ were similar in shape and size but the loops around the substrate binding cleft varied (Fig. 4a). Interestingly, although the bound oligomeric substrates for PyAly and A1-II’ had different chemical structures, their three-dimensional orientations were similar. As shown in Fig. 4a, both the position of the pyranose ring and the direction/orientation of the C-5 carboxyl group from each bound monomer were similar. These similarities between the two bound substrates was consistent with the fact described above that most of the PyAly residues functioning in subsites +1 to +3 were conserved in A1-II’ (Fig. 3). To describe this more clearly, we divided the substrate binding cleft into two sections using the cleavage site as a boundary: one section, named the “+” section, consisted of subsites +1, +2, +3, etc., and the other section, named the “-” section, consisted of subsites -1, -2, -3, etc. Therefore, the conserved residues resulted in similar “+” sections for PyAly and A1-II’, which further led to similar substrate binding profiles at the “+” subsites.

More interestingly, detailed interaction analysis suggested that the features of the “+” section derived from PyAly and A1-II’ were also applicable to the other PL7 alginate lyases. As described above, seven residues (underlined) from the three conserved regions QI(V)H, RXEL(V)R, and YFKXGXYXQ were involved in substrate binding at the “+” section. Notably, residues Q (from the first conserved region) and R (from the second conserved region) formed interactions with the C-5 carboxyl groups at subsites +1, +2, and +3, respectively, based on the structure of PyAly^H125A_Y223A^-M5 complex. Accordingly, PL7 alginate lyases tend to exhibit similar C-5 carboxyl group positions within the “+” sections. Since the positions of the C-5 carboxyl groups were “fixed”, the associated pyranose rings were restricted to specific spaces, resulting in similar positions. These similarities in the positions of the C-5 carboxyl groups and pyranose rings suggested that PL7 alginate lyases share similar substrate binding profiles at subsites +1, +2, and +3. Notably, these similarities are not related to the ability to recognize M/G units because the difference between the M and G units is difficult to discriminate by the overall substrate binding profile alone.

Next, the PyAly and AlyV structural comparisons focused on the “-” sections of the substrate binding clefts. At subsite -1, the three-dimensional position of the sugar unit in the AlyV^R91A^-M8 complex resembled that in the PyAly^H125A^-M8 complex (Fig. 4b). However, a clear angle was observed between the two sugar units at subsite -2; that is, the directions of the two C-5 carboxyl groups at subsite -2 were different. This observation was consistent with the fact that the PyAly residues involved in substrate binding in the “-” section are not conserved in AlyV (Fig. 3). In contrast to the “+” section, no conserved region was observed in the “-” section; thus, PL7 alginate lyases might exhibit diverse substrate binding profiles in their “-” sections.

In summary, we found an interesting structural feature in PL7 alginate lyases: the bound substrates in the binding clefts exhibited similar binding profiles in the “+” sections but distinct binding profiles in each “-” section. Combined with the biochemical characteristics of PyAly and AlyV, this structural feature was believed to be related to the differences in the Dp in the products. Accordingly, it was reasonable to propose that the different substrate binding profiles in the “-” sections of single-domain PL7 alginate lyases might determine product distributions, i.e., Dp specificity. Considering that some PL7 alginate lyases contain two or more domains and that additional domains might function during the substrate binding process, this speculation was limited to single-domain PL7 alginate lyases.

In a previous study, we reported a multidomain alginate lyase AlyB, which contained CBM32 (a carbohydrate binding domain) and a PL7 catalytic domain (named CD2^AlyB^)^24^. One intriguing feature of AlyB is that its CBM32 had little effect on enzymatic activity but rather specified that trisaccharides were predominant in the degradation products when these two domains were combined with an alpha helix linker. In contrast, CD2^AlyB^ generates oligomers with different Dps and shows little preference for trisaccharides. According to the speculation proposed above, the substrate binding profile of CD2^AlyB^ would change after the addition of CBM32 and the alpha helix linker, resulting in different product distributions for CD2^AlyB^ and AlyB. Thus, if the substrate binding profile of AlyB was determined, changes in the substrate binding profile could be observed intuitively, and the substrate binding profile/product distribution relationship would be clarified.

To explore the substrate binding profile of AlyB, we screened the crystals of the AlyB-substrate complex using the noncatalytic mutant AlyB^H360A_Y466A^ (His360 and Tyr466 are conserved catalytic residues) and the substrate G9 (GGGGGGGGG). Surprisingly, the structure of AlyB^H360A_Y466A^-G9 complex was solved at a resolution of 2.25 Å, binding the longest substrate among all solved alginate lyase structures and providing the substrate binding profile from subsite -3 to subsite +6 (Fig. 4c, Table 1). As expected, the structure of AlyB^H360A_Y466A^-G9 complex revealed the roles of CBM32 and the alpha helix linker in substrate binding. Structural comparison demonstrated that there was a clear linker twist that occurred upon G9 binding, which not only shortened the distance between CBM32 and CD2^AlyB^ but also enabled the ligand-binding region of CBM32 to turn toward the end of the “-” section of CD2^AlyB^, effectively blocking sugar extension. Analysis of the detailed interactions between AlyB and G9 indicated that a total of seven residues from CBM32 were involved in substrate binding, of which six residues formed hydrogen bonds (Fig. 4c) and Trp129 formed hydrophobic contact (Supplementary Fig. 5), and more interestingly, they all formed interactions with the sugar unit at subsite -3 (Fig. 4c). In contrast, only three residues from CD2^AlyB^ participated in substrate binding at subsites -2 and -1 (Fig. 4c). For substrate binding at the “+” section, the conserved residues in the PL7 family (corresponding to Arg289, Arg293 and Gln358 in AlyB) also formed interactions with the C-5 carboxyl group at subsites +1, +2 and +3, and a similar substrate binding profile was observed after structural comparison of the AlyB^H360A_Y466A^-G9 complex and the A1-II’-GGMG complex (Supplementary Fig. 6).

These results indicated that CBM32 functioned in substrate binding at the only “-” section and had little effect on substrate binding at the “+” section. Moreover, the extensive interactions formed between CBM32 and the sugar unit at subsite -3 played a critical role in determining the substrate binding profile at the “-” section. Additionally, the “-” section of CD2^AlyB^ accommodated exactly three sugar units because of the blocking effect produced by CBM32, which led to trisaccharide release during substrate degradation. Obviously, CD2^AlyB^ would exhibit a different substrate binding profile at the “-” section upon losing the interactions from CBM32, whereas a similar substrate binding profile at the “+” section would be displayed in the absence or presence of CBM32. The structure of AlyB^H360A_Y466A^-G9 complex provided structural evidence for different substrate binding profiles at the “-” section in one alginate lyase and confirmed the speculation that the substrate binding profile at the “-” section determines the product distributions of PL7 alginate lyases.

### Biochemical analysis of chimeric PyAly demonstrated that two loops determined the product distribution

**Fig. 5:**
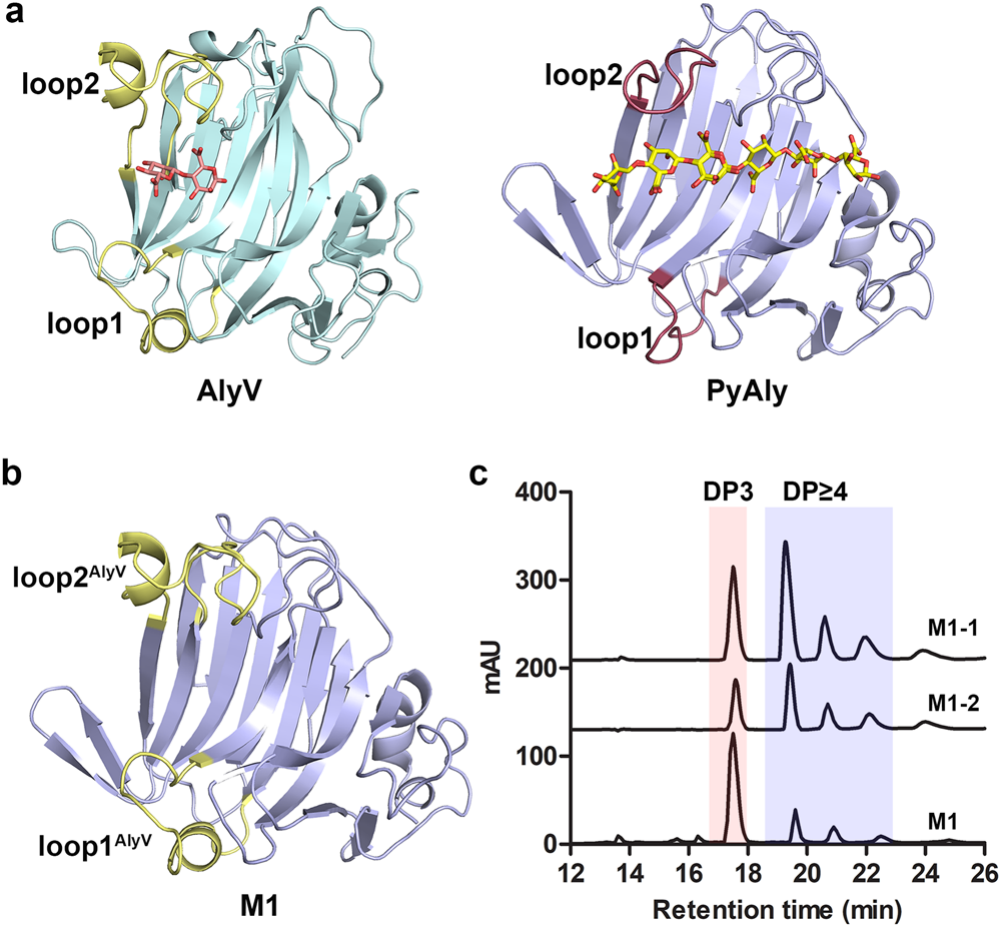
Two loops in the “-” section were demonstrated to determine product distribution. **a** The positions of two loops (loop1 and loop2) from AlyV and PyAly. loop1^AlyV^: Pro117-Ala134; loop2^AlyV^: Val183-Leu210; loop1^PyAly^: Thr108-Lys117; loop2^PyAly^: Arg159-Phe170. **b** Diagram of the constructed chimeric PyAly M1, containing loop1^AlyV^ and loop2^AlyV^. **c** Product distributions of M1, M1-1, and M1-2.

Since the “-” section had a great influence on the substrate binding profile, we continued to explore the structural elements that might lead to different substrate binding profiles in this section. Structural comparison of PyAly and AlyV demonstrated that the distinct differences in the “-” section were the lengths of two loops that linked four β-strands, here named loop1 and loop2 (Fig. 5a). For PyAly, loop1 consists of residues Thr108 to Lys117, and loop2 consists of residues Arg159 to Phe170. For AlyV, the corresponding two peptides linking the four β-strands were composed of residues Pro117 to Ala134 and residues Val183 to Leu210; thus, the structures of the two peptides of AlyV showed long loop insertions with small helices. Here, to simplify and standardize the description, the two peptides of AlyV were also named loop1 and loop2. As shown in Fig. 5a, the length of loop1 from AlyV was longer than that from PyAly. More interestingly, Lys131 from AlyV loop1 formed hydrophobic contacts with the sugar unit at subsite -2 (Supplementary Fig. 3), whereas PyAly loop1 was too short to form any interactions with the sugar unit at this subsite. In contrast to loop1, both AlyV loop2 and PyAly loop2 were involved in substrate binding at the “-” section, although AlyV loop2 was longer than PyAly loop2. The structural differences between loop1 and loop2 are supposed to regulate the substrate binding profiles in the “-” sections, leading to distinct product distributions.

To confirm the role of these two loops in product distribution, we designed three chimeric PyAly enzymes, M1-1, M1-2, and M1, prepared by replacing PyAly loop1/loop2 with AlyV loop1/loop2: M1-1 contained AlyV loop1 and PyAly loop2, M1-2 contained PyAly loop1 and AlyV loop2, and M1 contained AlyV loop1 and AlyV loop2 (Fig. 5b). The products generated by M1-1, M1-2 and M1 were analyzed using HPLC (Fig. 5c, Supplementary Table 2). Similar product distributions were observed for M1-1 and M1-2, which also resembled the product distribution generated by wild-type PyAly, indicating that a single loop replacement in PyAly had little influence on the substrate binding profile in the “-” section. In contrast, M1 exhibited a trisaccharide-predominant product distribution, which resembled that of AlyV rather than that of wild-type PyAly. This observation implied that the introduction of AlyV loop1 and loop2 into PyAly successfully changed its substrate binding at the “-” section, confirming that the product distribution was determined by the substrate binding profile at the “-” section.

**Fig. 6:**
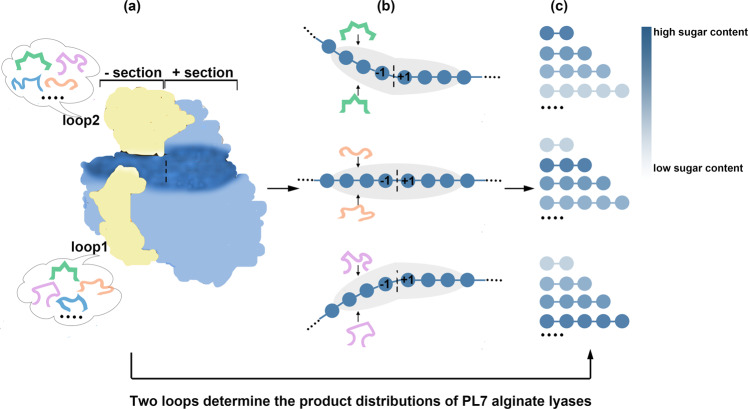
The relationship between two loops, the substrate binding profile and product distribution. The monomer unit of alginate is shown in a blue circle. The endolytic PL7 alginate lyase **a** degraded long-chain alginate **b** into a mixture of oligosaccharides with different Dps **c**. The oligosaccharides are colored with a gradient from white (low sugar content) to blue (high sugar content), indicating different product distributions. Our study demonstrated that the “+” sections of the substrate binding clefts of PL7 alginate lyases contained several conserved residues, leading to similar substrate binding profiles. In contrast, the “-” sections of the substrate binding clefts exhibited diversity, resulting in different substrate binding profiles. Thus, the overall substrate binding profiles of PL7 alginate lyases were mainly determined by the “-” sections. Structural and biochemical analyses indicated that the diverse substrate binding profiles were attributed to the different loops (loop1 and loop2) around the “-” section. Accordingly, these two loops determined the substrate binding profiles of PL7 alginate lyases. Because the substrate binding profile directly influenced the product distribution, we associated the two loops with the product distributions. Thus, two loops in the “-” section determined product distributions.

Then, the relationship of two loops (loop1 and loop2), substrate binding profile and product distribution was revealed as follows. (a) The lengths and sequences of the two loops influenced substrate binding at the “-” section specifically; therefore, variations in these two loops led to diverse substrate binding profiles at the “-” section, which was in marked contrast to the similar substrate binding profiles at the “+” section (Fig. 6). (b) The substrate binding profile directly influenced the product distribution; therefore, the different product distributions were attributed to diverse substrate binding profiles at the “-” section (Fig. 6). Based on the conclusions from (a), the structure/function relationship between the two loops and the product distribution was revealed, that the length and sequence of the two loops determined product distribution, i.e., Dp specificity. Furthermore, the Dp specificity/loop relationship could be extended to other single-domain PL7 alginate lyases as a general pattern because of the conserved β-jelly roll fold and three conserved regions (Fig. 6).

### Analysis of the reported PL7 alginate lyase structures suggested that the length of loop1 could be used to classify endolytic single-domain PL7 alginate lyases

According to the conclusion that two loops in the “-” section determine the product distributions of single-domain PL7 alginate lyases, we continued to explore how these two loops affect product distributions. To address this question, more results on loop sequence and product distribution relationships are needed. Therefore, the 12 reported PL7 alginate lyase structures were screened based on the CAZy database (May 2021), and three multimodular alginate lyase structures (PDB: 5XNR^22^, 3ZPY^23^, and 5ZU5^24^) and one exolytic alginate lyase structure (PDB: 4BE3^23^) were excluded. The remaining eight endolytic single-domain alginate lyase structures were selected for examination (PDB: 2ZAA^27^, 1UAI^21^, 1VAV^20^, 5Y33^25^, 4OZX, 5ZQI, 7C8F/7C8G^26^, and 6YWF). However, high sequence identity was found between alginate lyases AlyC3 (PDB: 7C8G) and AlyV (58.3%) and between alginate lyases Psalg7A (PDB: 6YWF) and PyAly (45.1%). Therefore, AlyC3 and Psalg7A were excluded from the following studies, while the genes encoding the other six alginate lyases were synthesized and expressed to obtain recombinant proteins for product analysis. Moreover, the sequences of the two loops of the six alginate lyases were obtained based on structural comparisons of the six alginate lyases with AlyV/PyAly (Table 2).

**Table 2:** The loop sequences of eight PL7 alginate lyases with solved structures.

Protein	PDB ID	loop1	loop2	Ref.
AlyV	7W16	PINPRASVANTDKAKNDA	VIKNNAVNCGSKSGNKGTEECKNAYLKL	This study
AlyA	4OZX	HVTTTGVNWQVGR	HEPRKGFGDEQ	—
PyAly	7W13/7W18	TLPTPLRGMK	RLNNKSGDAGRF	This study
A1-II’	2ZAA	QLPSSGKI	KQNSDGTGSD	ref. ^27^
AlgAT5	5ZQI	HLPEVKDH	GDGE	—
ALY-1	1UAI	HLPEVKPH	KGDD	ref. ^21^
FlAlyA	5Y33	ISKEPDGKYSRV	LKDLNAPYKEMLSEHAW	ref. ^25^
PA1167	1VAV	AVPSTRR	VRERPDDGGTR	ref. ^20^

Considering that alginate lyases might exhibit different substrate specificities, the preferred substrate should be determined and used for product analysis. In addition, the product distributions used for Dp comparison analysis should be defined because the product distribution of an alginate lyase using an endolytic action mode might vary in terms of the degradation process. The alginate degradation process can be roughly divided into three stages: (1) the initial stage, where the alginate substrate is degraded slightly and the long-chain substrate is the main component; (2) the middle stage, where the alginate substrate is continuously degraded and oligomeric products increasingly accumulate; and (3) the final stage, where the alginate substrate is totally degraded into oligomeric products, and these oligomers might be further degraded if more enzyme is supplied or the reaction time is prolonged. In the final stage, loop1 and loop2 might not function in substrate binding if the oligomeric substrate is too short to occupy the whole substrate binding cleft. Accordingly, the product distributions of the middle stage could be used to determine the enzyme preference for the Dp of the products toward long-chain substrates (including not only the initial long-chain substrate but also the long-chain intermediate products that were further degraded as substrates) and reflect the functions of loop1 and loop2. Therefore, the substrate specificity of the six alginate lyases mentioned above was determined first using alginate, polyM and polyG as substrates, and the preferred substrate was used in the following product distributions assay. Next, the alginate substrate degradation process for each alginate lyase was determined, and the product distribution presented at the end of the middle stage was compared with that from other alginate lyases (see Methods for more detailed information).

**Fig. 7:**
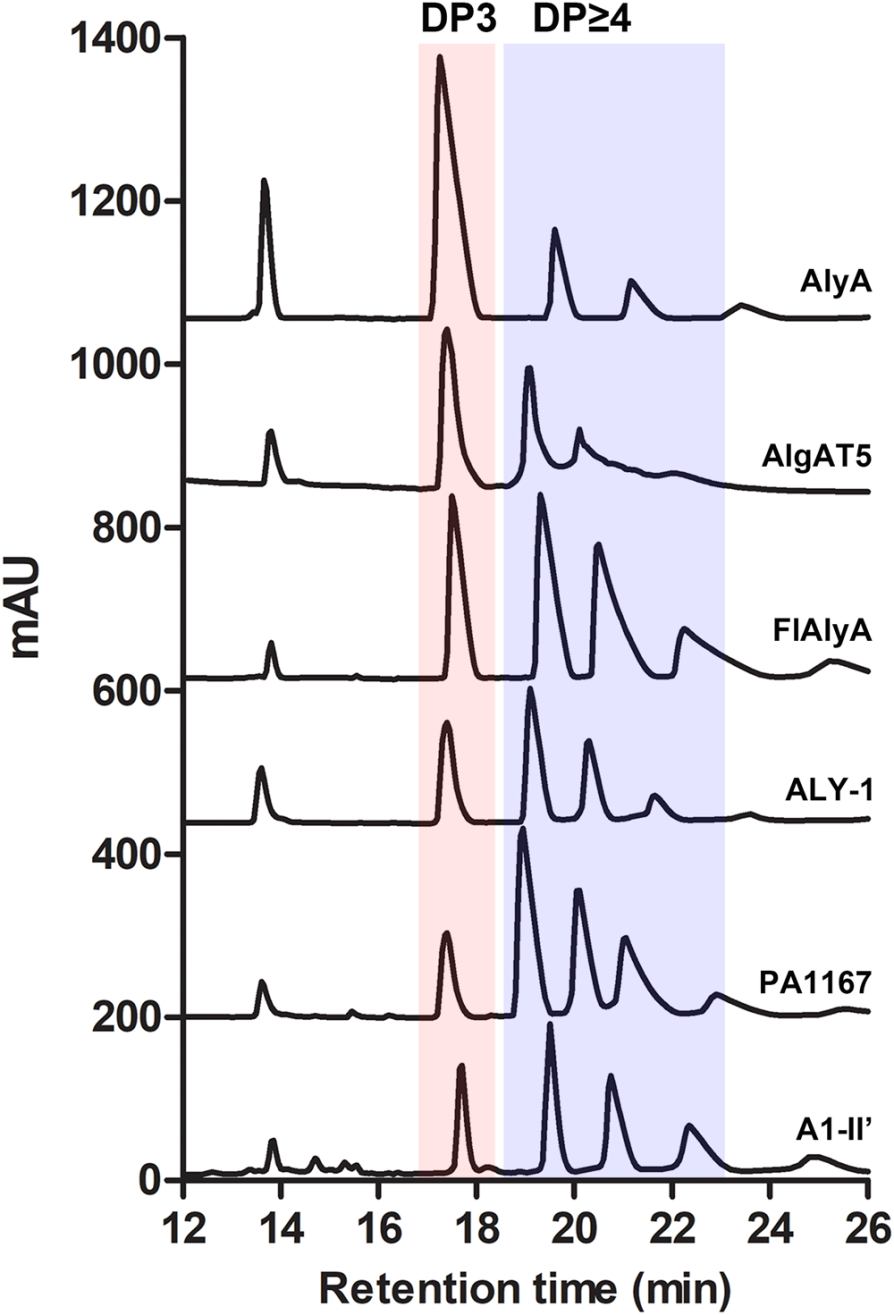
The products generated by six PL7 alginate lyases were analyzed by HPLC. The  main products of these alginate lyases were determined by the quantity of each oligomer.

As shown in Fig. 7 and Supplementary Table 2, all six alginate lyases generated oligomeric products ranging from Dp2 to Dp6; however, they could be divided into two groups based on the quantity of each oligomer. Among the six alginate lyases, AlyA (PDB: 4OZX) preferentially generated trisaccharides (61.1%) but also generated a small amount of oligomeric products with Dp > 3; however, the other five alginate lyases preferentially generated larger products, especially tetrasaccharides. Interestingly, the product distribution of AlyA resembled that of AlyV, and the product distributions of the other five alginate lyases resembled that of PyAly. According to the product distributions results, the loops were also divided into two groups, and their lengths were further compared because of their low sequence similarity. However, no regular pattern was observed in the loop2 lengths (Table 2). In contrast, the lengths of loop1 from both AlyV and AlyA were longer than those from PyAly and the other five alginate lyases mentioned above (Table 2), which could be connected to the difference in product distributions. Therefore, the Dp specificity/loop relationship could be further simplified as follows: loop1 length shows a good negative correlation with product length.

### A detailed association between loop1 length and product distribution was determined

**Fig. 8:**
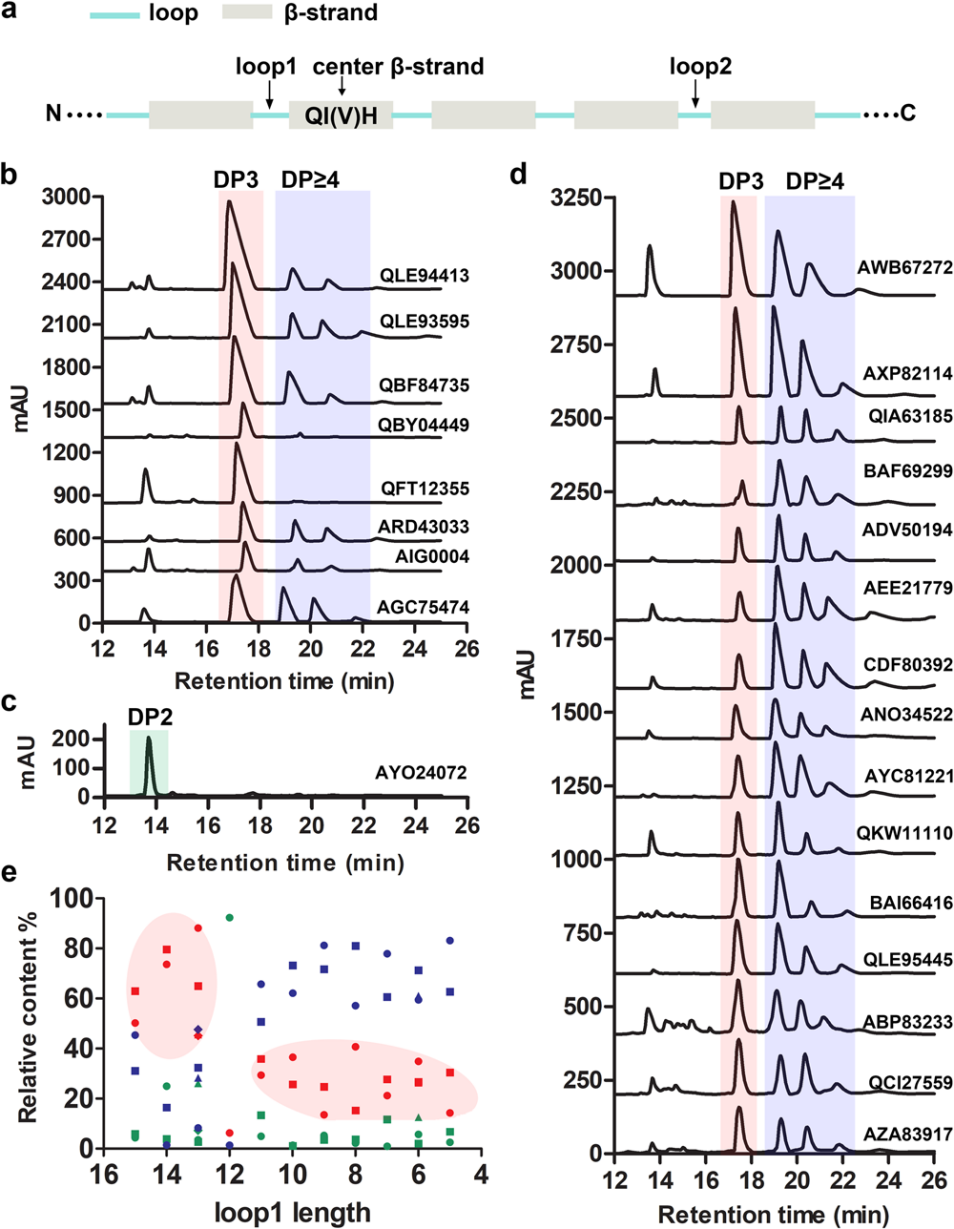
The association between loop1 length and product distribution was determined. **a** The approach to determine the positions of loop1 and loop2 for single-domain PL7 alginate lyases. Based on the similarities in product distributions, the 24 annotated-only PL7 alginate lyases were classified into three groups and are presented in **b**–**d**. **e** The association between loop1 length and product distribution. The disaccharides, trisaccharides, and oligosaccharides (DP ≥ 4) are shown in green, red and blue, respectively. The circles, squares, rhombi, and triangles represent different alginate lyases sharing the same loop1 length.

To utilize the length of loop1 for product distribution prediction and alginate lyase classification, the question of how to determine the loop1 sequence for annotated-only PL7 alginate lyases should first be addressed. Clearly, it is difficult to determine which peptides fold into loop1 and loop2 when the three-dimensional structure of the alginate lyase is unknown. However, the structural features of PL7 alginate lyases, the β-jelly roll fold containing mainly β-strands and loops in the secondary structure, suggest that the secondary structure positions might help determine the sequences of loop1 and loop2. Because the conserved region QI(V)H is located in a β-strand, here named the center β-strand, the position of this β-strand could be determined through sequence alignment. Once the center β-strand is defined, the general spatial positions of the other β-strands and the loops linking two neighboring β-strands could be determined based on the conserved β-jelly roll fold. In brief, the secondary structures of one annotated-only PL7 alginate lyase could be predicted using the online JPred server (http://www.compbio.dundee.ac.uk/jpred/), and the resulting center β-strand containing the conserved region QI(V)H could be used as a position marker to determine which loop corresponds to loop1 or loop2 and then to determine the sequences of loop1 and loop2. As shown in Fig. 8a, loop1 was adjacent to the N-terminal end of the center β-strand, and loop2 was located behind the C-terminal end of the center β-strand with two β-strands and two loops inserted between loop2 and the center β-strand. Thus, a unique approach to locate the two loop positions of these annotated-only PL7 alginate lyases was established, which successfully bridged the protein primary sequence to its structure.

Next, we established a database (Supplementary Data 1) containing the loop1 information for all annotated-only single-domain PL7 alginate lyases, which enabled further exploration to confirm the relationship between the length of loop1 and product distribution. The loop1 sequences of 1421 annotated-only PL7 alginate lyases were determined, and the lengths of loop1 ranged from 4 to 15 residues (Supplementary Data 1). Based on the length of loop1, the alginate lyases were classified into 12 groups. For each group, at least one alginate lyase was randomly selected as a representative combined with the sequence alignment. In total, 27 alginate lyase genes were randomly selected and synthesized, of which 24 were successfully expressed in *E. coli* BL21(DE3), resulting in recombinant proteins (Supplementary Table 5). Therefore, product distributions analyses for these 24 alginate lyases were carried out, including substrate specificity analysis and substrate degradation process analysis (see Methods for more detailed information).

Similar to the six alginate lyases with solved structures, the 24 annotated-only alginate lyases could be classified into two groups by comparing their product distributions with those determined from AlyV and PyAly. More importantly, the loop1s in the group similar to AlyV were longer than that in the group similar to PyAly, which further confirmed the conclusion that loop1 length could be used to predict the product distribution of an alginate lyase. As shown in Fig. 8b, trisaccharides were predominant in the products of QBF84735 (loop1 = 15), QLE94413 (loop1 = 14), QFT12355 (loop1 = 14), QLE93595 (loop1 = 13) and QBY04449 (loop1 = 13), with measured trisaccharide contents of 63.0%, 79.6%, 73.6%, 65.0% and 88.2%, respectively (Supplementary Table 2). Additionally, the main products of ARD43033 (loop1 = 15), AGC75474 (loop1 = 13) and AIG00004 (loop1 = 13) were determined to be trisaccharides because the trisaccharide contents of 50.2%, 45.1% and 45.6% were clearly higher than the contents of the other oligomers (Supplementary Table 2). These observations suggested that alginate lyases containing long loop1s (loop1 length ≥13) preferentially generate trisaccharides. In contrast, the trisaccharide contents decreased considerably for the alginate lyases containing short loop1s (loop1 length ≤11), and the contents of larger oligomers (Dp≥4) increased correspondingly (Fig. 8d, e, Supplementary Table 2). Notably, the dominant products of AYO24072 (loop1 = 12) were disaccharides (92.3%) but not trisaccharides (Fig. 8c, Supplementary Table 2), which indicated that some alginate lyases might preferentially generate disaccharides; this observation should be taken into account during product distribution prediction. Notably, a longer loop1 is also required for the generation of disaccharides because the disaccharide contents for the alginate lyases with short loop1s did not exceed 14%.

Based on the association between loop1 length and the product distribution described above, the product distribution prediction criteria for annotated-only PL7 alginate lyases were summarized as follows: alginate lyases with long loop1s (loop1 length ≥12) tended to generate trisaccharides (high probability) or disaccharides, while alginate lyases with short loop1s (loop1 length ≤11) tended to generate larger oligomers (Dp ≥ 4) (Fig. 8e). This loop1 length/product distribution relationship will facilitate the classification of endolytic PL7 alginate lyases.

## Conclusion

In this study, we have shown that two loops (loop1 and loop2) of single-domain PL7 alginate lyases located in the “-” section of the substrate binding cleft are the determinants for product distributions after performing biochemical and structural research on AlyV, PyAly, and AlyB. Furthermore, the Dp specificity/loop relationship was further simplified, as the product distribution could be predicted by the length of loop1 alone, as shown by additional analyses of PL7 alginate lyases with solved structures. Moreover, a unique approach to predict the two loops of annotated-only PL7 alginate lyases was proposed, which successfully bridged the protein primary sequence to its structure, enabling additional analyses of the PL7 alginate lyase database without the determination of three-dimensional structures. An additional database study showed an association between the length of loop1 and product distribution, providing a method to predict product distributions for annotated-only PL7 alginate lyases. These findings facilitate the understanding of the role of an annotated-only PL7 alginate lyase in alginate degradation and oligosaccharide preparation.

## Materials and methods

### Materials

Alginate (M/G ratio: 0.6) was purchased from Shanghai Yuanye Co., Ltd. (Shanghai, China). PolyG and polyM were prepared by acid hydrolysis of alginate^24^. Briefly, the mixture containing alginate (2% (w/v)) and HCl (1 M) was incubated at 90 °C for 6 h. The resulting precipitate was collected and dissolved in the NaHCO_3_ solution (8% (m/v)). Then, the pH of the mixture was adjusted to 2.85 and 1.0 to precipitate polyG and polyM, respectively. Saturated M5, M8, and G9 were purchased from Qingdao BZ Oligo Biotech Co., Ltd. (Qingdao, China).

### Gene cloning, mutagenesis, expression and protein purification

The AlyV and PyAly genes were amplified from the genomic DNA of *Vibrio pelagius* and *Pyropia yezoensis*, respectively. The cloned genes were ligated into the pET28a and pET32a vectors using *Bam*H I and *Xho* I sites as the restriction enzyme sites, respectively. The recombinant plasmids were transformed into *E. coli* BL21(DE3), cultured at 37 °C until the OD_600_ reached 0.6-0.8 and then induced by 0.2 mM isopropyl-β-D-thiogalactopyranoside (IPTG). After overnight incubation at 16 °C, the cells were harvested by centrifugation, suspended in Tris-HCl buffer (20 mM, pH 8.0) containing 200 mM NaCl, lysed by sonication and then clarified by centrifugation. The resulting supernatant was purified using affinity chromatography, and the fused tag was cleaved by a protease. For crystallographic studies, AlyV/PyAly and the mutants were further purified over a Sephacryl-S-100 column that had been previously equilibrated with 20 mM Tris-HCl, pH 7.5, containing 200 mM NaCl after affinity purification. Protein concentrations were determined by measuring the absorption at 280 nm and using the molar extinction coefficients calculated by the online ExPASy server.

Site-directed AlyV, PyAly, and AlyB mutants were generated using the QuikChange® site-directed mutagenesis method (Agilent Technologies) and verified by DNA sequencing. Then, these mutants were expressed and purified as described above. The primers used in this study are listed in Supplementary Table 6.

The genes of six alginate lyases with solved structures and 27 annotated-only alginate lyases were synthesized (Sangon Biotech) and expressed in *E. coli* BL21(DE3). The PDB ID/gene accession numbers and plasmids used for protein expression are listed in Supplementary Table 5. Except for AEU36217 (loop1 length of 4), QLI64135 (loop1 length of 4), and QHJ13020 (loop1 length of 12), the other genes were expressed successfully, and the resulting recombinant proteins were collected from cell lysates for product distributions analysis.

### Crystallization, data collection, and structure refinement

AlyV^R91A^, PyAly^H125A^, PyAly^H125A_Y223A^, and AlyB^H360A_Y466A^ were concentrated to 10–18 mg/mL before screening. For cocrystallization, substrates M5, M8, and G9 were added to the inactive mutants at a 10:1 molar ratio to form an enzyme-substrate complex. Crystallization conditions were manually screened against the Screen I, Screen II (Hampton Research) and JCSG + (Qiagen) kits using the hanging drop vapor diffusion method. Briefly, crystallization reagent (250 µL) was added into the reservoir, and the complex sample (1 µL) and reagent (1 µL) were mixed in the cover slide. Crystals were initially obtained at 15 °C after approximately 2–3 days, and after optimization, the AlyV^R91A^-M8 complex was crystallized in 1.6 M sodium citrate tribasic dihydrate (pH 6.5). The PyAly^H125A^-M8 complex was crystallized in 0.1 M imidazole (pH 7.0), 20% Jeffamine® ED-2001 and 5% glycerinum. The PyAly^H125A_Y223A^ -M5 complex was crystallized in 0.1 M HEPES sodium (pH 7.5) with 10% 2-propanol and 20% PEG4000. The AlyB^H360A_Y466A^-G9 complex was crystallized in 0.3 M Li_2_SO_4_, 0.1 M Tris-HCl (pH 8.0) and 35% PEG4000. All crystals were picked up and cryoprotected by soaking in mother solution containing 10% glycerol for a few seconds and then flash-cooled in liquid nitrogen.

X-ray diffraction data were collected on the BL18U1 and BL19U1 beamlines at the Shanghai Synchrotron Radiation Facility (SSRF) and processed using HKL2000^32^. The initial structure solutions were obtained by molecular replacement using *Phaser*^33^, and the templates used for AlyV^R91A^, PyAly^H125A^, PyAly^H125A_Y223A^ and AlyB^H360A_Y466A^ were as follows: the modeled structures of AlyV and PyAly generated by the on-line Swiss-model server^34^, and the determined structure of AlyB (PDB: 5ZU5)^24^. The overall structures were then refined by *REFMAC5*^35^ in combination with manual model building in *Coot*^36^. The final structure figures were generated using *CCP4mg*^37^ and *PyMOL* (https://pymol.org), and the final structures will be deposited in the Protein Data Bank (PDB: 7W13, 7W18, 7W16, 7W12; Table 1).

### Preparation of chimeric PyAly

To evaluate the roles of the two loops in product distribution, three chimeric PyAly enzymes were designed. The three chimeric PyAly enzymes were named M1-1, M1-2 and M1: M1-1 contained AlyV loop1 and PyAly loop2, M1-2 contained PyAly loop1 and AlyV loop2, and M1 contained AlyV loop1 and AlyV loop2. To prepare M1-1, M1-2 and M1, the loop1^AlyV^ and loop2^AlyV^ fragments were first amplified from the AlyV gene. Moreover, the pMCSG9 plasmids containing the PyAly gene were linearized to delete loop1^PyAly^ or loop2^PyAly^, resulting in two truncated plasmids: plasmid 1 (lacking loop1^PyAly^) and plasmid 2 (lacking loop2^PyAly^). Then, with the ClonExpress® II One Step Cloning Kit (Vazyme), the loop1^AlyV^ fragment was inserted into linearized plasmid 1 to construct the M1-1 gene, and the loop2^AlyV^ fragment was inserted into linearized plasmid 2 to construct the M1-2 gene. Next, the loop2^PyAly^ fragment of M1-1 gene was substituted with loop2^AlyV^ fragment to construct the plasmid with the M1 gene. The resulting three plasmids were verified by DNA sequencing. Next, the M1-1, M1-2, and M1 genes were expressed and purified for product distributions analyses.

### Enzymatic activity assay

Alginate lyase activity was determined by measuring the increase in absorbance at 235 nm due to the release of unsaturated uronic acid for 20 min at 37 °C. Reaction mixtures (200 µL) were composed of 0.2% (m/v) substrate in 20 mM Tris-HCl buffer (pH 8.0) containing 500 mM NaCl. Three substrates, alginate, polyG and polyM, were used to determine the substrate specificities of AlyV, PyAly, and the 30 selected alginate lyases. One unit of enzyme activity was defined as an A235 increase of 1 per minute.

### Product analysis

Based on the results of the substrate specificity assay, the preferred substrate (alginate/polyM/polyG) for each alginate lyase was used for product analysis. The reaction mixtures (500 μL) contained enzymes and substrate (2 mg/mL) dissolved in 20 mM Tris-HCl buffer (pH 8.0) and 200 mM NaCl. Because the enzymes exhibited different enzymatic activities, the amount of each enzyme used in the degradation reactions was different. Fifty microliters of purified AlyV (1.20 mg/mL), PyAly (0.87 mg/mL), M1-1 (2.28 mg/mL), M1-2 (2.32 mg/mL) and M1 (8.9 mg/mL) were used. For the 30 selected alginate lyases (six characterized alginate lyases and 24 annotated-only alginate lyases), crude proteins were used, and the volumes of the proteins ranged from 50 μL to 200 μL. The products generated at different reaction times were prepared by heating the reaction mixtures at 100 °C for 5 min, and the resulting products were analyzed by thin-layer chromatography (TLC) developed with n-butanol/formic acid/water (4:6:1, v/v/v) and visualized by diphenylamine/aniline/phosphate^24^. Through the TLC analyses, the product distributions as a function of degradation time were determined. Based on these results, the three stages of the degradation process were determined. Here, the TLC result of alginate lyase AXP82114 is provided as an example to illustrate the determination of the middle stage (Supplementary Fig. 7). The product that was generated close to the end of the middle stage was prepared again and analyzed by HPLC. Products were loaded onto a YMC-Pack ODS-AM/S column (5 μm/12 nm) connected to an UltiMate 3000 system (Thermo Scientific). The column was eluted with acetonitrile containing 5 mM tetrabutylammonium bromide (TBAB) at a flow rate of 1 mL/min, and the absorbance was monitored at 230 nm.

### Prediction of the two loops for the annotated-only PL7 alginate lyases

Considering that the determined Dp specificity/loop relationship is specific to single-domain PL7 alginate lyases acting in endo-type mode, the annotated-only PL7 alginate lyases were screened before protein secondary structure prediction. Two types of annotated-only PL7 alginate lyases were excluded from the following loop analysis: 1) those that might contain two or more domains, i.e., those with longer sequences (here, a length of 400 residues was set as the threshold for screening) and 2) those that shared similar sequences with the exolytic alginate lyase AlyA5 (PDB: 4BE3)^23^. After screening, the resulting alginate lyases were submitted to JPred to predict their secondary structures^38^. In combination with sequence alignment, the conserved QI(V)H region-containing β-strand was identified. Then, the sequences of loop1 and loop2 were determined (Fig. 8a). For alginate lyases containing more than one QI(V)H region, additional sequence alignment was performed to accurately find the conserved QI(V)H region.

### Statistics and reproducibility

All experiments were repeated at least three times with similar results. GraphPad Prism 5 was used for data analysis. Data were presented as means ± standard deviation (SD), and *n* = 3 biologically independent experiments.

### Reporting summary

Further information on research design is available in the Nature Research Reporting Summary linked to this article.

## Supplementary information

Supplementary Information

Description of Additional Supplementary Files

Supplementary Data 1

Supplementary Data 2

Reporting Summary

## Data Availability

All data generated or analyzed during this study are included in this published article (and its supplementary information files). The sequences of PyAly, AlyV, and AlyB have been deposited in GeneBank with accession codes: BAI66416.1, OM743979, and WP_116871942, respectively (https://www.ncbi.nlm.nih.gov/genbank/). The structures of PyAly^H125A^-M8, PyAly^H125A_Y223A^-M5, AlyV^R91A^-M8, and AlyB^H360A_Y466A^-G9 complexes have been deposited in the Protein Data Bank (https://www.wwpdb.org), with PDB: 7W13, 7W18, 7W16, and 7W12, respectively. A database is provided in Supplementary Data 1. Source data for the main figures are supplied as Supplementary Data 2. All other data are available from the corresponding author on reasonable request.
