# Supplementary material for: Determination of oligosaccharide product distributions of PL7 alginate lyases by their structural elements
Source: Commun Biol. 2022 Aug 2;5:782. doi: 10.1038/s42003-022-03721-1 (PMC9345997; doi:10.1038/s42003-022-03721-1)
Supplement: Supplementary file 1 — Supplementary Information [file 42003_2022_3721_MOESM1_ESM.pdf]

Determination of oligosaccharide product distributions of PL7 alginate lyases by their structural elements

Keke Zhang[1,2], Zhijian Li[1], Qiaoyun Zhu[1], Huansheng Cao[3], Xinxin He[1], Xiao-Hua Zhang[1], Weizhi Liu[1,2*], Qianqian Lyu[1, 2*]

[1]MOE Key Laboratory of Marine Genetics and Breeding, College of Marine Life Sciences, Ocean University of China, Qingdao 266003, China.

[2]Laboratory for Marine Biology and Biotechnology, Pilot National Laboratory for Marine Science and Technology, Qingdao, 266237, China

[3]Division of Natural and Applied Sciences, Duke Kunshan University, 8 Duke Ave, Kunshan 215316, China.

*Correspondence e-mail: lyuqianqian@ouc.edu.cn, liuweizhi@ouc.edu.cn

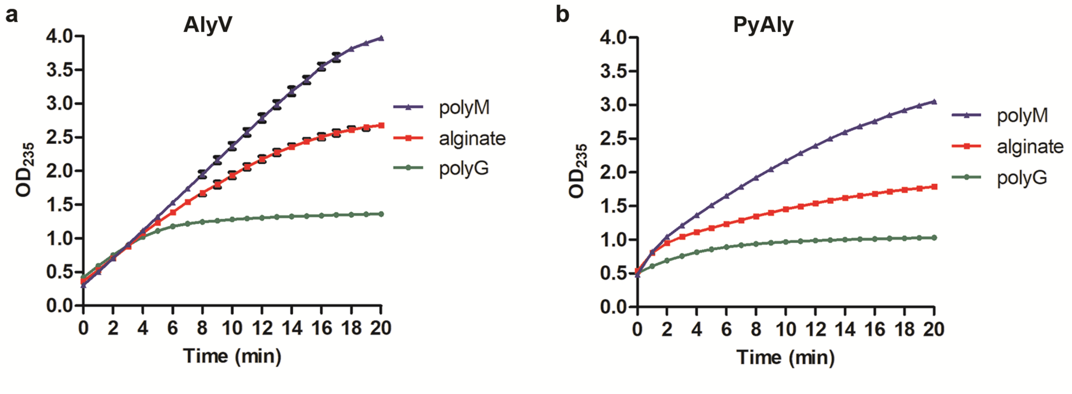

Supplementary Figure 1 Substrate specificities of AlyV and PyAly.

Data were presented as means ± SD. n = 3 biologically independent experiments.

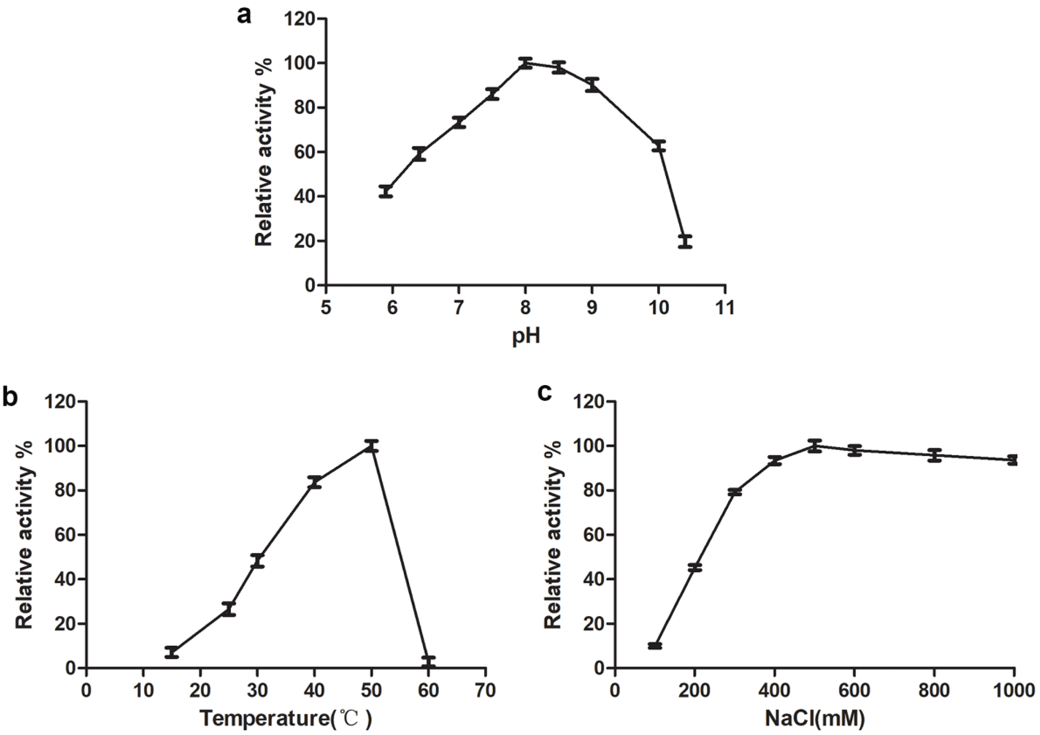

Supplementary Figure 2 Effects of pH (a), temperature (b) and NaCl concentration (c) on the AlyV activities.

To measure the effect of pH, different buffers were used, including 50 mM Na_2HPO_4-NaH_2PO_4 (pH 6.0, 6.4, 7 and 7.5), 50 mM Tris-HCl (pH 8.0, 8.5 and 9.0) and 50 mM Na_2CO_3-$NaHCO_3$ (pH 10.0 and 10.4). The effects of temperatures and NaCl concentration on the AlyV activities were measured in Tris-HCl (pH 8.0) buffers. Alginate was used as substrate.

Data were presented as means ± SD. n = 3 biologically independent experiments.

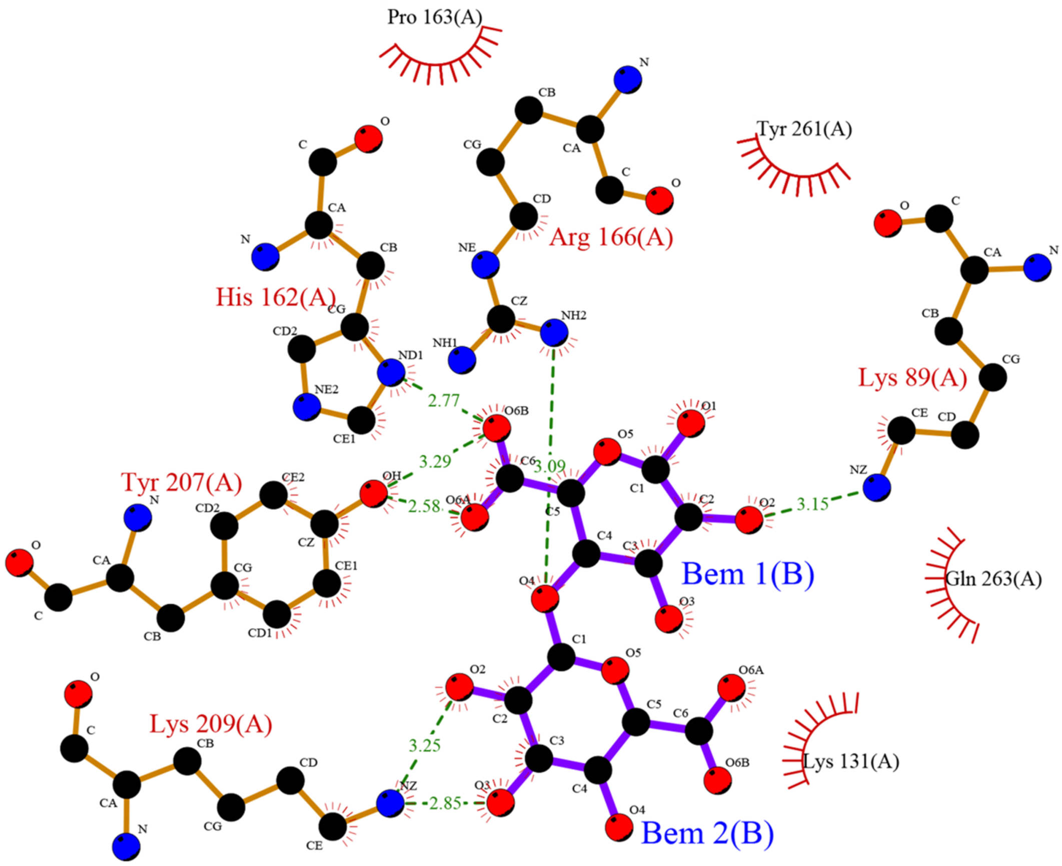

Supplementary Figure 3 ***Ligplot*** **of interactions between AlyV^{R91A} and M8.** The interactions shown are those mediated by hydrogen bonds and by hydrophobic contacts. Hydrogen bonds are indicated by dashed lines between the atoms involved, while hydrophobic contacts are represented by an arc with spokes radiating towards the ligand atoms they contact. The contacted atoms are shown with spokes radiating back. The Bem represents β-D-mannuronic acid (M) unit.

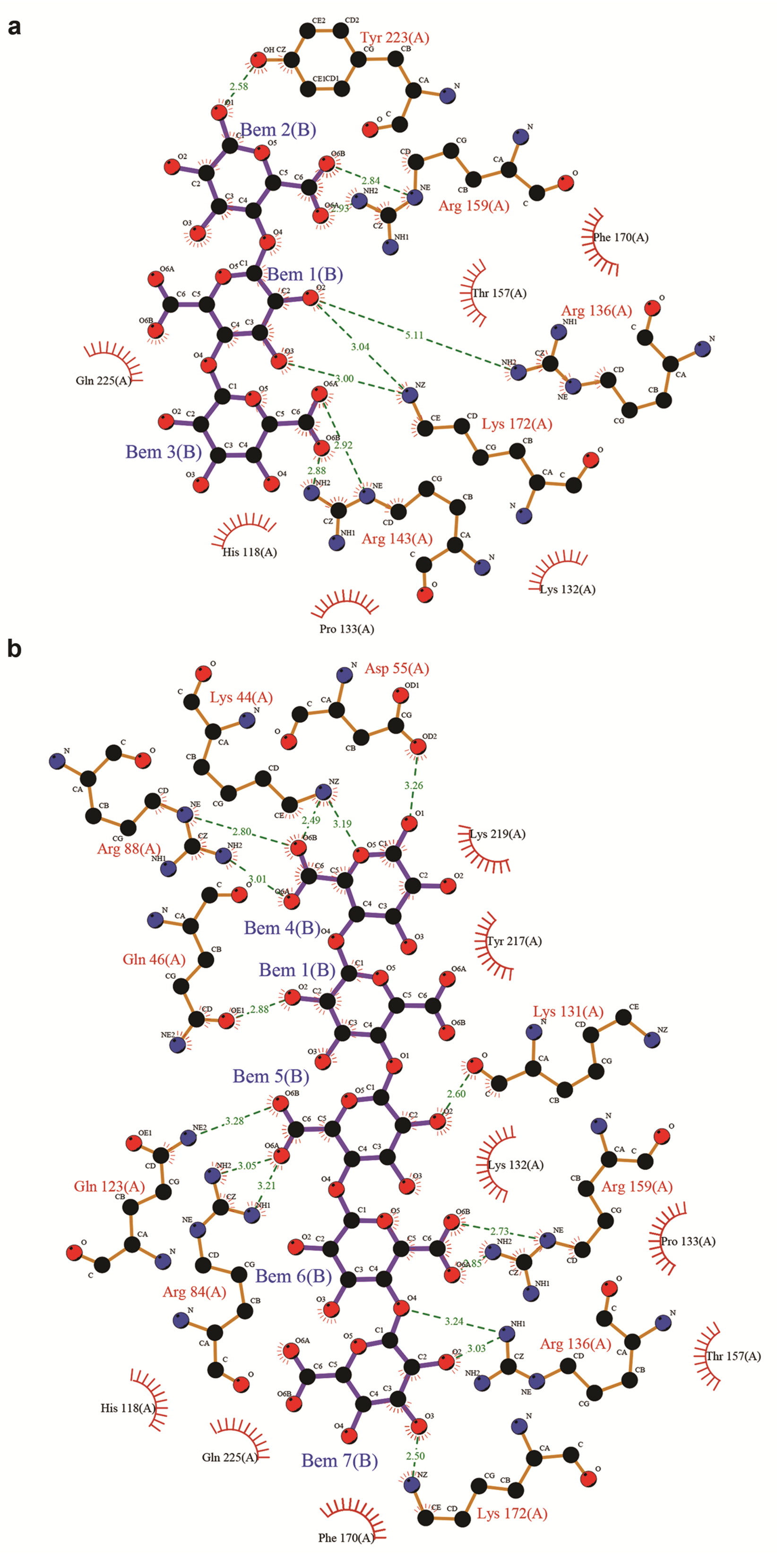

Supplementary Figure 4 ***Ligplot*** **of interactions between PyAly^{H125A} and M8 (a), and interactions between PyAly^{H125A_Y223A} and M5 (b).**

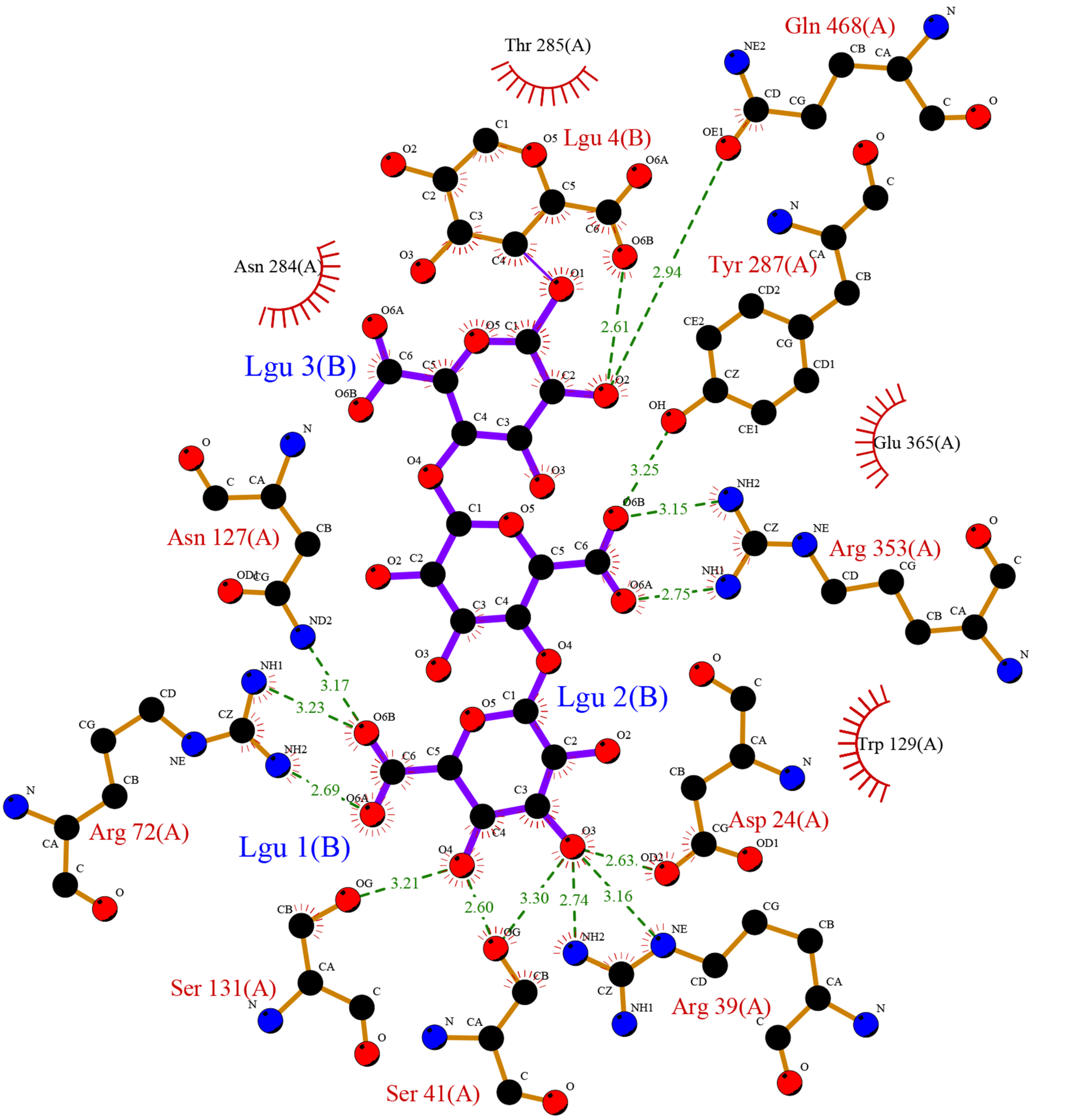

Supplementary Figure 5 ***Ligplot*** **of interactions between AlyB$^{\mathrm{H360A_Y466A}}$ and G9 at subsites -3 to -1.**

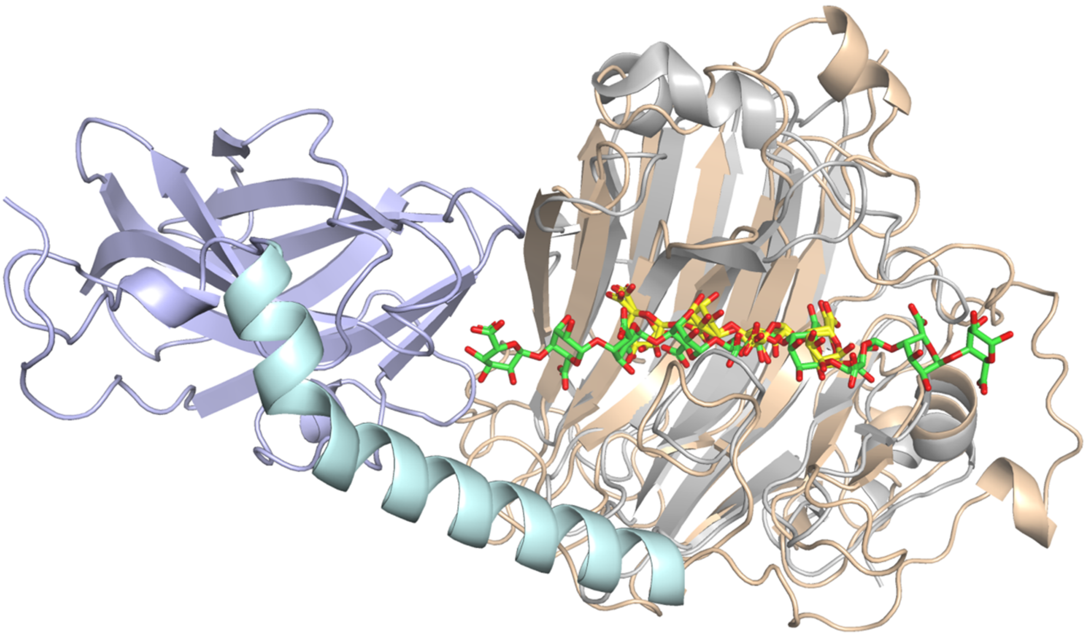

Supplementary Figure 6 Structural comparison of the AlyB^{H360A_Y466A}-G9 and A1-II'- GGMG complexes.

AlyB^{H360A_Y466A} is shown as cartoon in light blue, palecyan and wheat, corresponding to CBM32, α-helix linker and CD2^{AlyB}. A1-II' is shown in gray. The G9 and GGMG are presented in sticks in green and yellow, respectively.

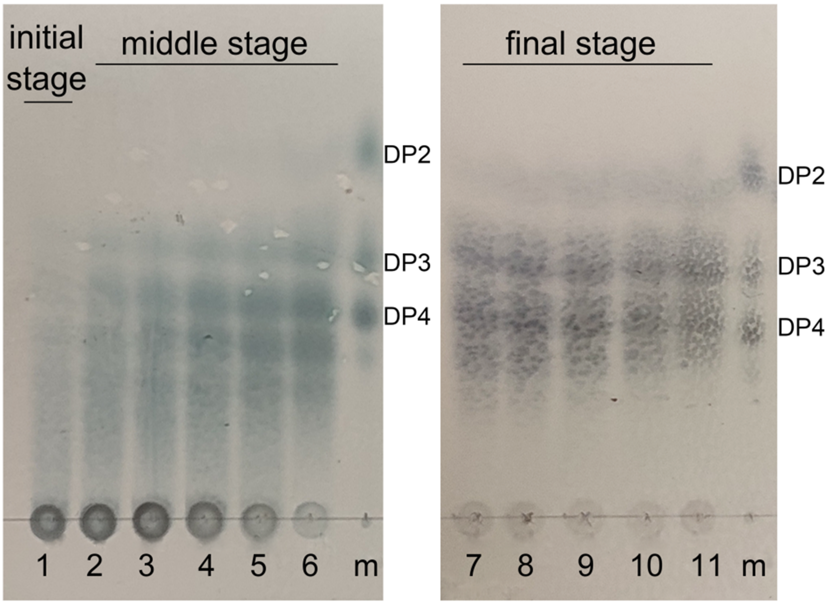

Supplementary Figure 7 The alginate degradation process of alginate lyase AXP82114.

Based on the features described above, the amount of the long-chain substrate can be used as the criterion to divide the whole degradation process into three stages. Due to their high molecular weights, long-chain substrates cannot be separated by TLC, and instead remain at the origin. Thus, the amount of long-chain substrate can be estimated by the color at the origin (if the color changes from dark to light, this indicates that the amount of long-chain substrate decreased). A large amount of long-chain substrate and few oligosaccharides were observed in lane 1, indicating that the degradation process was in the initial stage. Lanes 2-6 show that the amount of long-chain substrate decreased continuously along with increasing amounts of oligosaccharides, indicating that the degradation process was in the middle stage. Only oligosaccharides were observed in lanes 7-11, indicating that the degradation process was in the final stage. After determination of the middle stage, the following question was which reaction timepoint during this stage should be selected for product preparation. The reaction timepoint close to the end of the middle stage was easy to identify by TLC analysis, and the product

generated at this timepoint seldom contained long-chain substrates, which facilitated subsequent HPLC analysis. Therefore, the product close to the end of the middle stage (here, 1 h for alginate lyase AXP82114) was prepared for Dp specificity comparison. Lane m: unsaturated oligosaccharides (DP2, DP3, and DP4). Lanes 1−11: the degradation products collected at 1 min, 5 min, 10 min, 20 min, 30 min, 1 h, 3 h, 5 h, 9 h, 11 h and 24 h, respectively.

Supplementary Table 1 Kinetic parameters of AlyV using alginate as substrate.

V_{max} (μM/min)	K_m (mg/mL)	K_{cat} (s^{-1})
176.70	3.35	28.59

The kinetic parameters were calculated based on the nonlinear regression software GraphPad Prism 5.0. V_{max}: maximum rate, K_m: michaelis constant, K_{cat}: catalytic constant.

Supplementary Table 2 The loop1 length and product distributions of PL7 alginate lyases.

Protein	Substrate	Product distribution/Relative content %					
		DP2	DP3	DP4	DP5	DP6	DP7
AlyV	polyM	2.06	82.90	10.36	4.25	0.42	--
PyAly	polyM	--	21.87	44.81	15.28	13.76	4.29
M1	polyM	2.51	67.76	13.6	8.66	5.43	2.03
M1-1	polyM	--	23.92	31.12	16.38	16.74	11.83
M1-2	polyM	0.54	27.53	36.13	14.30	13.40	8.09
AlyA	polyG	14.74	61.11	12.73	7.58	3.84	--
FlAlyA	polyM	2.09	22.05	27.03	27.84	16.04	4.94
ALY-1	polyG	10.30	24.36	31.23	20.56	9.89	3.67
AlgAT5	polyG	7.09	32.07	24.24	26.82	9.79	--
A1-II’	Alginate	3.80	16.73	29.06	24.78	18.06	7.57
PA1167	Alginate	4.07	12.62	33.00	21.24	21.53	7.53

Protein	Loop1 length	Substrate	Product distribution/Relative content %					
			DP2	DP3	DP4	DP5	DP6	DP7
QBF84735	15	polyM	5.96	62.95	24.17	5.72	1.20	--
ARD43033	15	Alginate	4.41	50.19	21.80	18.75	4.84	--
QLE94413	14	polyM	3.91	79.62	10.76	4.98	0.73	--
QFT12355	14	polyG	24.95	73.59	1.46	--	--	--
AGC75474	13	Alginate	7.27	45.11	26.02	17.84	3.76	--
AIG00004	13	polyM	26.12	45.59	16.75	8.83	2.71	--
QLE93595	13	Alginate	2.68	64.97	12.50	11.62	6.87	1.36
QBY04449	13	polyG	3.54	88.16	7.02	1.27	--	--
AYO24072	12	Alginate	92.29	6.32	1.39	--	--	--
AWB67272	11	Alginate	13.36	35.9	28.34	18.3	4.10	
AXP82114	11	Alginate	4.92	29.37	35.79	22.44	6.50	0.97
QIA63185	10	polyG	1.11	25.67	23.99	26.56	16.60	6.08
QLE95445	10	polyG	1.35	36.52	33.24	19.02	8.59	1.27
ANO34522	9	polyG	3.55	24.73	34.99	21.86	14.87	--
AEE21779	9	Alginate	5.29	13.52	28.84	19.87	21.67	10.82
CDF80392	8	Alginate	3.70	15.28	33.96	18.19	21.45	7.43
BAI66416	8	polyM	2.21	40.67	41.66	10.35	5.11	--
ABP83233	7	Alginate	11.69	27.68	23.56	18.19	15.22	3.67
AYC81221	7	polyM	0.93	21.18	30.39	26.67	13.99	6.85
QKW11110	6	Alginate	12.58	26.39	34.46	14.08	7.92	4.57
ADV50194	6	polyG	2.00	26.73	36.34	22.59	12.34	--
AZA83917	6	Alginate	5.74	34.84	23.69	22.64	8.91	4.17
QCI27559	5	Alginate	6.82	30.49	21.53	24.11	11.37	5.68
BAF69299	5	polyM	2.56	14.32	36.71	25.99	14.53	5.89

Supplementary Table 3 The hydrogen bonds formed between AlyV^{R91A} and two sugar units.

Subsite	Sugar atom	Amino acid	Distance (Å)
-2	O2	Lys209/NZ	3.25
	O3	Lys209/NZ	2.85
-1	O2	Lys89/NZ	3.15
	O6A	Tyr207/OH	2.58
	O6B	Tyr207/OH	3.29
		His162/ND1	2.77
	O4	Arg166/NH2	3.09

Supplementary Table 4 The hydrogen bonds formed between PyAly and six sugar units.

Subsite	Sugar atom	Amino acid	Distance (Å)
-3	O6A	Arg143/NE	2.92
	O6B	Arg143/NH2	2.88
-2	O2	Arg136/NH2	2.90
		Lys172/NZ	3.04
	O3	Lys172/NZ	3.00
-1	O1	Tyr223/OH	2.58
	O4	Arg136/NH1	3.24
	O6A	Arg159/NH2	2.93
	O6B	Arg159/NE	2.84
+1	O2	Lys131/O	2.60
	O6A	Arg84/NH1	3.21
		Arg84/NH2	3.05
	O6B	Gln123/NE2	3.28
+2	O2	Gln46/OE1	2.88
+3	O1	Asp55/OD2	3.26
	O6A	Arg88/NH2	3.01
	O6B	Arg88/NE	2.80
		Lys44/NZ	2.49
	O5	Lys44/NZ	3.19

Supplementary Table 5 The accession numbers, PDB IDs, protein expression vectors and strains used in this study.

Accession number	PDB ID	Plasmid	Strain
Q59478	4OZX	pET28a	*E.coli* BL21(DE3)
KM008909	5Y33	pColdII	*E.coli* BL21(DE3)
NC_003076.8	1UAI	pET28a	*E.coli* BL21(DE3)
A0A4V8GZK9	5ZQI	pET28a	*E.coli* BL21(DE3)
BK023103	2ZAA	pColdII	*E.coli* BL21(DE3)
NP_249858.1	1VAV	pET28a	*E.coli* BL21(DE3)
QBF84735	--	pET28a	*E.coli* BL21(DE3)
ARD43033	--	pET28a	*E.coli* BL21(DE3)
QLE94413	--	pET28a	*E.coli* BL21(DE3)
QFT12355	--	pET28a	*E.coli* BL21(DE3)
AGC75474	--	pET28a	*E.coli* BL21(DE3)
AIG00004	--	pET28a	*E.coli* BL21(DE3)
QLE93595	--	pET28a	*E.coli* BL21(DE3)
QBY04449	--	pET28a	*E.coli* BL21(DE3)
AYO24072	--	pET28a	*E.coli* BL21(DE3)
AWB67272	--	pET28a	*E.coli* BL21(DE3)
AXP82114	--	pET28a	*E.coli* BL21(DE3)
QIA63185	--	pET28a	*E.coli* BL21(DE3)
QLE95445	--	pET28a	*E.coli* BL21(DE3)
ANO34522	--	pET28a	*E.coli* BL21(DE3)
AEE21779	--	pET28a	*E.coli* BL21(DE3)
CDF80392	--	pET28a	*E.coli* BL21(DE3)
BAI66416	--	pET28a	*E.coli* BL21(DE3)
ABP83233	--	pET28a	*E.coli* BL21(DE3)
AYC81221	--	pET28a	*E.coli* BL21(DE3)
QKW11110	--	pMCSG9	*E.coli* BL21(DE3)
ADV50194	--	pET28a	*E.coli* BL21(DE3)
AZA83917	--	pMCSG9	*E.coli* BL21(DE3)
QCI27559	--	pET28a	*E.coli* BL21(DE3)
BAF69299	--	pET28a	*E.coli* BL21(DE3)
AEU36217	--	pET28a/pMCSG9	*E.coli* BL21(DE3)
QLI64135	--	pET28a/pMCSG9	*E.coli* BL21(DE3)
QHJ13020	--	pET28a/pMCSG9	*E.coli* BL21(DE3)

E. coli: *Escherichia coli*

Supplementary Table 6 Primers used in this study.

Protein	Primers
AlyV	F: 5'-CGGGATCCGCAAATGCTTCAGATAAGGCTGCTC-3'
	R: 5'-CCGCTCGAGTTAACGAATTACTGGCTCGCTTTCTAC-3'
PyAly	F: 5'-CGGGATCCGCCCGTCGTCCGCC-3'
	R: 5'-CCGCTCGAGTTAGCTAACGGTCAGACCG-3'
AlyV^{R91A}	F: 5'-GCCTGGTTACAAAAATGCGAGTGAAGTACGCATCTATAAAAAC-3'
	R: 5'-GTTTTTATAGATGCGTACTTCACTCGCATTTTTGTAACCAGGC -3'
AlyV^{Q139A}	F: GATGCGATCACATACCTTGCGGTACATAATGCGGGTAGTG
	R: CACTACCCGCATTATGTACCGCAAGGTATGTGATCGCATC
AlyV^{H141N}	F: 5'-CACATACCTTCAAGTAAACAATGCGGGTAGTGTCTCTGC-3'
	R: 5'-GCAGAGACACTACCCGCATTGTTTACTTGAAGGTATGTG-3'
AlyV^{Y261F}	F: 5'-GCTATTTCAAAGCCGGTGTTTTTAACCAGTTTAAGAACGG-3'
	R: 5'-CCGTTCTTAAACTGGTTAAAAACACCGGCTTTGAAATAGC-3'
PyAly^{H125A}	F: 5'-CTGGATGCAGGTTGCCGGCGGTAGCAAAGGC-3'
	R: 5'-GCCTTTGCTACCGCCGGCAACCTGCATCCAG-3'
PyAly^{Y223A}	F: 5'-CAAAGCGGGTGTTGCCGTTCAGGAAGGTAGCCCGG-3'
	R: 5'-CCGGGCTACCTTCCTGAACGGCAACACCCGCTTTG-3'
AlyB^{H360A}	F: 5'-CATTGGTCAGATTGCCGATCAAAACGATGAGCCG
	R: 5'-CGGCTCATCGTTTTGATCGGCAATCTGACCAATG
AlyB^{Y466A}	F: 5'-CAAAGCGGGTGTTGCCAACCAAAATATCAGCGGC
	R: 5'-GCCGCTGATATTTTGGTTGGCAACACCCGCTTTG
Loop1^{AlyV}	V-1-F: 5'-gtcgtatggtgggcgttctgCCAATCAACCCACGCGCG-3'
	V-1-R: 5'-acctgcatccaggtgaagtgCGCATCATTCTTCGCTTTGTC-3'
Loop2^{AlyV}	V-2-F: 5'- acaccatgctggcgaccgttGTAATTAAGAACAATGCTGTGAACTGTG-3'
	V-2-R: 5'-gtacccagaacgatctttttTAGTTTCAAATACGCATTTTTACACTCT-3'
M1-1	Linearized vector：
	P-1-F: 5'-CACTTCACCTGGATGCAGGTTC-3'
	P-1-R: 5'-CAGAACGCCCACCATACGAC-3'
M1-2	Linearized vector：
	P-2-F: 5'-AAAAAGATCGTTCTGGGTACCCG-3'
	P-2-R: 5'-AACGGTCGCCAGCATGGT-3'
