# Supplementary material for: Determination of oligosaccharide product distributions of PL7 alginate lyases by their structural elements
Source: Commun Biol. 2022 Aug 2;5:782. doi: 10.1038/s42003-022-03721-1 (PMC9345997; doi:10.1038/s42003-022-03721-1)
Supplement: Supplementary file 2 — Description of Additional Supplementary Files [file 42003_2022_3721_MOESM2_ESM.pdf]

Description of Additional Supplementary Files

File name: Supplementary Data 1
Description: The database contains the loop information of all annotated-only singledomain PL7 alginate lyases.

File name: Supplementary Data 2
Description: The source data behind the graphs in the paper.
